# Autophagy and cell wall integrity pathways coordinately regulate the development and pathogenicity through MoAtg4 phosphorylation in *Magnaporthe oryzae*

**DOI:** 10.1371/journal.ppat.1011988

**Published:** 2024-01-30

**Authors:** Pusheng Guo, Yurong Wang, Jiayun Xu, Zhixiang Yang, Ziqi Zhang, Jinyi Qian, Jiexiong Hu, Ziyi Yin, Leiyun Yang, Muxing Liu, Xinyu Liu, Gang Li, Haifeng Zhang, Ryan Rumsey, Ping Wang, Zhengguang Zhang

**Affiliations:** 1 Department of Plant Pathology, College of Plant Protection, Nanjing Agricultural University, and Key Laboratory of Integrated Management of Crop Diseases and Pests, Ministry of Education, Nanjing, China; 2 The Key Laboratory of Plant Immunity, Nanjing Agricultural University, Nanjing, China; 3 Department of Microbiology, Immunology, and Parasitology, Louisiana State University Health Sciences Center, New Orleans, Louisiana, United States of America; Purdue University, UNITED STATES

## Abstract

Autophagy and Cell wall integrity (CWI) signaling are critical stress-responsive processes during fungal infection of host plants. In the rice blast fungus *Magnaporthe oryzae*, autophagy-related (ATG) proteins phosphorylate CWI kinases to regulate virulence; however, how autophagy interplays with CWI signaling to coordinate such regulation remains unknown. Here, we have identified the phosphorylation of ATG protein MoAtg4 as an important process in the coordination between autophagy and CWI in *M*. *oryzae*. The ATG kinase MoAtg1 phosphorylates MoAtg4 to inhibit the deconjugation and recycling of the key ATG protein MoAtg8. At the same time, MoMkk1, a core kinase of CWI, also phosphorylates MoAtg4 to attenuate the C-terminal cleavage of MoAtg8. Significantly, these two phosphorylation events maintain proper autophagy levels to coordinate the development and pathogenicity of the rice blast fungus.

## Author summary

Autophagy is a conserved cellular degradation and recycling process important in maintaining proper cellular homeostasis whereas the cell wall integrity (CWI) signaling pathway is also a conserved regulatory pathway in stress response. CWI signaling not only operates alone but also functions through crosstalk with autophagy during fungal infection of host plants. Despite the importance, little is known about how autophagy and CWI signaling coordinate their regulatory roles. Here, we have found that autophagy-related (ATG) protein MoAtg4 phosphorylation mediates the coordination between autophagy and CWI signaling, which underlies the development and pathogenicity of *Magnaporthe oryzae*. Both ATG protein MoAtg1 and CWI kinase MoMkk1 phosphorylate MoAtg4, which inhibits the deconjugation and C-terminal cleavage of MoAtg8, respectively, to maintain proper autophagy levels. Our study revealed a novel coordination between autophagy and CWI signaling and how the two pathways coordinate to regulate the development and pathogenicity of the blast fungus.

## Introduction

Autophagy is a highly conserved catabolic process that mediates intracellular component degradation through the autophagosomes to maintain cellular homeostasis in response to cellular and environmental stresses [1,2], which can be categorized into nonselective and selective autophagy based on its selectivity for substrates [3]. Nonselective autophagy is critical for pathogenicity, whereas selective autophagy (e.g., pexophagy and mitophagy) is dispensable for appressorium-mediated host infection in the rice blast fungus *Magnaporthe oryzae* [4–6]. In the Baker’s yeast *Saccharomyces cerevisiae*, the autophagosome formation correlates well with the amount of autophagy-related (ATG) protein 8 (Atg8)-phosphatidylethanolamine (PE), the lipidation form of Atg8 [7–9]. At the same time, the cysteine protease Atg4 cleaves the C-terminus of the newly synthesized cytoplasmic Atg8 to produce an active variant that undergoes lipidation modification and further conjugates PE of the phagophore assembly site (PAS) [10,11]. Once autophagosome forms at PAS, Atg4 deconjugates Atg8 from the Atg8-PE anchor on the autophagosome membrane, a critical process for Atg8 recycling and subsequent autophagosome fusion with vacuole [12,13]. Studies in *S*. *cerevisiae* also found that Atg4-mediated deconjugation of Atg8-PE is inhibited by ULK/ULK protein kinase Atg1-dependent phosphorylation, ensuring Atg8 anchoring until autophagosome formation [1].

The cell wall integrity (CWI) signaling pathway is critical for maintaining strong but elastic cell wall during growth and development and also in responses to the external stress [14–16]. In *S*. *cerevisiae*, CWI signals stimulate the small G protein Rho1 that binds to the protein kinase C (Pkc1) to activate the conserved mitogen-activated protein kinase (MAPK) cascade, composed of Bck1, Mkk1/2, and Slt2/Mpk1, which then targets the transcription factors Rlm1 and the SBF complex (Swi4 and Swi6) to govern the expression of cell wall biosynthesis and cell cycle genes, respectively [17–19]. Many plant pathogenic fungi, including *M*. *oryzae*, *Fusarium graminearum*, and *Claviceps purpurea*, utilize this conserved signaling pathway to balance the cell wall homeostasis, which is critical for development and pathogenicity [20–23]. In *M*. *oryzae*, deletions of the CWI MAPK cascade components *MoMCK1*, *MoMKK1*, or *MoMPS1* lead to abnormal chitin distribution and virulence attenuation [22,24,25]. Recent studies have also shown that deletions of these kinases result in aberrant autophagy by unknown mechanisms [16].

As the critical processes in response to environmental stresses, connections between autophagy and CWI signaling have been previously examined in *S*. *cerevisiae* and *M*. *oryzae* [16,26–28]. In *S*. *cerevisiae*, Slt2 promotes mitophagy by affecting mitochondrial recruitment to the PAS, and also regulates pexophagy without affecting nonselective autophagy [26–28]. In *M*. *oryzae*, endoplasmic reticulum (ER) stress induced by abnormal protein synthesis activates MoAtg1-dependent MoMkk1 phosphorylation to enhance CWI signaling, thus promoting infection [16]. At the same time, CWI signaling addresses cell wall stress by targeting protein production, implying a possible important relationship between the CWI signaling pathway and nonselective autophagy (hereafter “autophagy”). However, the underlying mechanism of how autophagy coordinats with CWI signaling to regulate development and pathogenicity is unknown. Here, we revealed that two phosphorylation events of MoAtg4, MoMkk1-mediated phosphorylation in the cytoplasm and MoAtg1-mediated phosphorylation at PAS, collectively maintain proper autophagy and cellular homeostasis.

## Results

### Cell wall stress-induced autophagy is dependent on CWI kinases in *M*. *oryzae*

**Fig 1:**
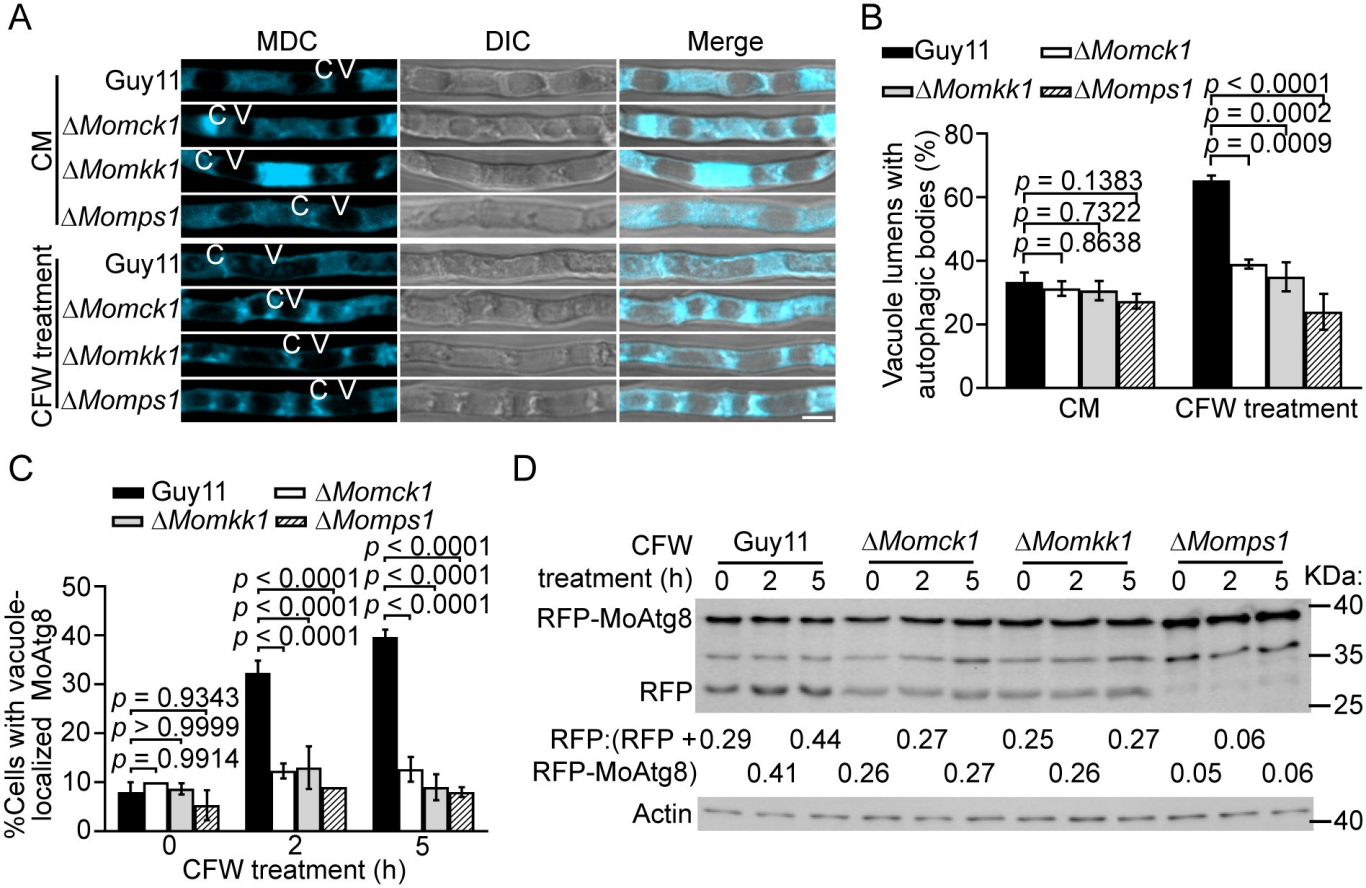
Cell wall stress-induced autophagy is dependent on CWI kinases. (A) Hyphae of Guy11, Δ*Momck1*, Δ*Momkk1*, and Δ*Momps1* strains cultured in CM were treated without or with CFW for 5 h (both adding 2 mM PMSF), and autophagic bodies (AB) were observed by confocal microscopy after MDC staining. C: cytoplasm. V: vacuole. (B) Quantification of vacuoles with AB as shown in (A). (C) Quantification of cells with vacuole-localized RFP-MoAtg8 after CFW treatment. (D) Total mycelial proteins after CFW treatment were extracted and analyzed by Western blot analysis with anti-RFP and anti-Actin antibodies. The amount of free RFP was compared with the total amount of RFP-MoAtg8 and free RFP to quantify autophagic levels. CFW treatment: treated with 1 mg/ml CFW. Data (n = 100) from three independent experiments were used for statistical analysis by two-way ANOVA with Tukey’s HSD. Scale bar: 5 μm.

To explore the coordination between autophagy and the CWI signaling pathway, we treated the wild-type strain Guy11 and CWI kinase mutant stains Δ*Momck1*, Δ*Momkk1*, and Δ*Momps1* with cell wall stressor Calcofluor White (CFW) and performed autophagic body (AB) staining using monodansylcadaverine (MDC) [29–31]. Vacuoles with AB were significantly increased after 5 h of treatment in Guy11 (~65%), but not in the mutants (Fig 1A and 1B). In addition, the expression of the autophagic marker protein RFP-MoAtg8 in these strains revealed that vacuole-localized RFP was significantly increased in Guy11, but not in the mutants (Fig 1C). This result was confirmed by Western blotting analysis that showed an elevated ratio of free RFP in Guy11 in comparison to the mutants (Fig 1D). We also used sodium dodecyl sulfate (SDS) as a second stressor and observed a similar result (S1A–S1C Fig). In addition, the degradation of MoSec63-GFP, an ER-phagy marker of *M*. *oryzae* [32], was detected under CFW treatment. These results showed that cell wall stress did not induce ER-phagy (S1D Fig). Together, these results indicate that cell wall stress-induced autophagy is dependent on CWI kinases in *M*. *oryzae*.

### CWI kinase MoMkk1 interacts with and phosphorylates MoAtg4 in the cytoplasm

**Fig 2:**
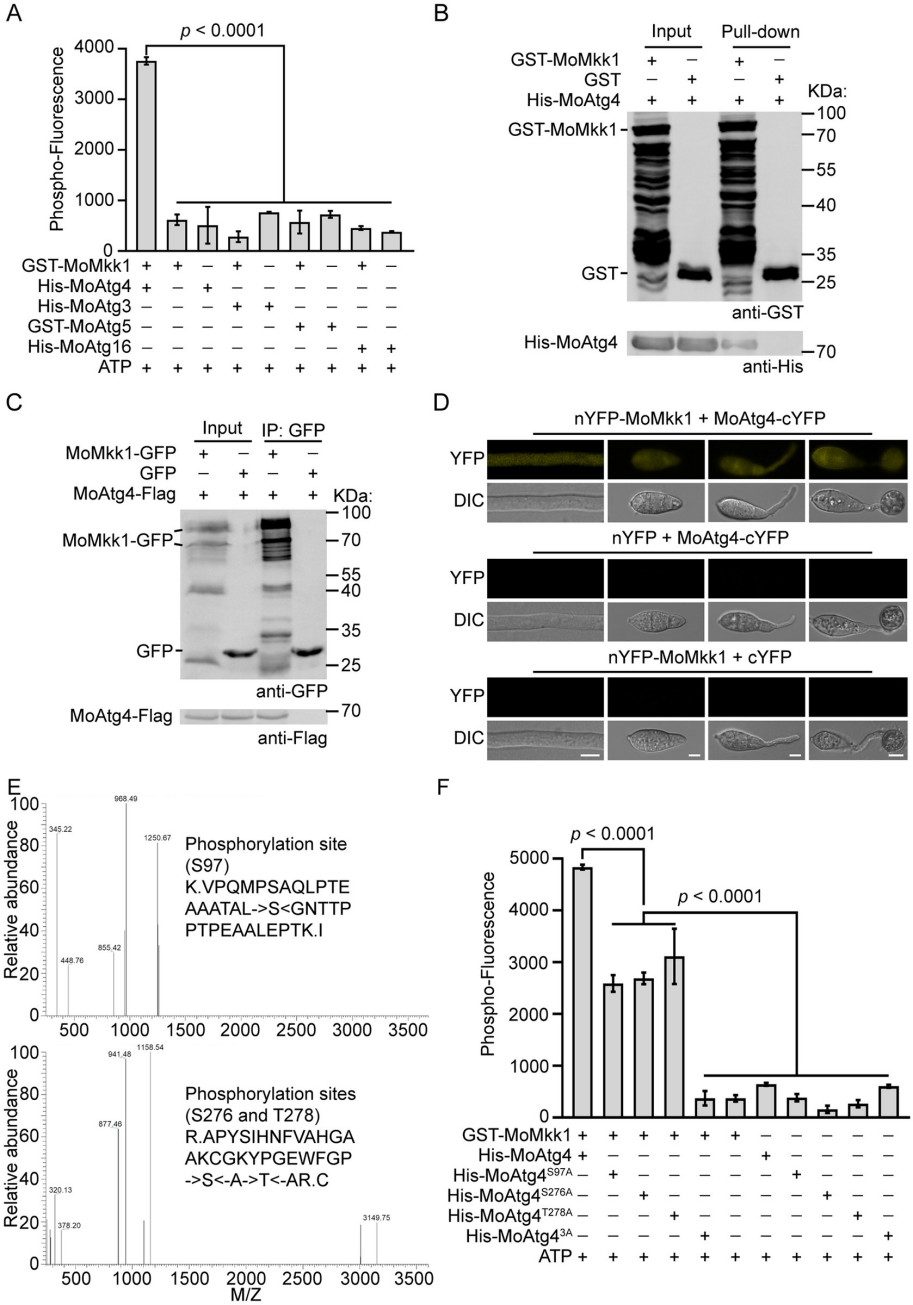
The CWI kinase MoMkk1 interacts with and phosphorylates MoAtg4 in the cytoplasm. (A) *In vitro* phosphorylation reaction of GST-MoMkk1 with His-MoAtg4, His-MoAtg3, GST-MoAtg5, or His-MoAtg16 fusion proteins in the presence of 50 μM ATP, and phosphorylation proteins were stained using Pro-Q Diamond Phosphorylation Gel Stain. Cytation3 microplate reader was then used to measure the Phospho-Fluorescence signal at 590 nm (excited at 530 nm). (B) GST-MoMkk1, empty GST, and His-MoAtg4 proteins were obtained for GST pull-down analysis. (C) MoMkk1-GFP and MoAtg4-Flag were co-expressed in the Guy11 strain, and proteins were extracted for co-IP assay by Western blot analysis using anti-GFP and anti-Flag antibodies. Proteins from Guy11 co-expressing GFP and MoAtg4-Flag were used as the control. (D) BiFC observation in the strain co-expressing MoMkk1-nYFP and MoAtg4-cYFP during various developmental stages. The strains co-expressing empty nYFP with MoAtg4-cYFP and MoMkk1-nYFP with empty cYFP were used as controls. Scale bar: 5 μm. (E) Peptides of MoMkk1-dependent MoAtg4 phosphorylation were identified by LC-MS/MS analysis under CFW treatment. (F) GST-MoMkk1, His-MoAtg4, His-MoAtg4^S97A^, His-MoAtg4^S276A^, His-MoAtg4^T278A^, and His-MoAtg4^3A^ fusion proteins were obtained for *in vitro* phosphorylation analysis. 3A: replaced S97, S276, and T278 with A to mimic nonphosphorylated form of MoAtg4. Data from three independent experiments were used for statistical analysis by one-way ANOVA with Tukey’s HSD.

Previous studies identified 22 ATG proteins that are essential for autophagy in *M*. *oryzae* [6], among which several were found to interact with MoMkk1 via yeast-two-hybrid (Y2H) screening, including MoAtg3, 4, 5, and 16 that are directly involved in Atg8 lipidation [16]. In addition, MoMkk1 mediates the crosstalk between the CWI signaling pathway and autophagy under ER stress [16]. We, therefore, hypothesized that autophagy coordinates CWI signaling through direct phosphorylation of ATG proteins by MoMkk1. To test this hypothesis, *in vitro* phosphorylation assays were performed using phosphoprotein gel staining [16,33,34], and the results showed significantly increased phospho-fluorescence only between MoMkk1 and MoAtg4, but not MoAtg3, 5, or 16 (Fig 2A). This result suggested that MoMkk1 can phosphorylate MoAtg4. Consequently, we focused on the interaction between MoMkk1 and MoAtg4.

We next examined the localizations of MoMkk1 and MoAtg4 in *M*. *oryzae*. Both were localized to the cytoplasm with or without cell wall stress, though MoAtg4 was also accumulated at PAS that was marked with MoApe1-RFP (S2A and S2B Fig). CFW treatment did not affect the numbers of cells with PAS-localized MoAtg4 (S2C Fig). These results implied a direct interaction between MoMkk1 and MoAtg4. To test the physical interaction between the two, GST pull-down and co-immunoprecipitation (co-IP) assays were performed that showed positive interaction (Fig 2B and 2C). Furthermore, a bimolecular fluorescence complementation (BiFC) assay confirmed that the interaction occurs in the cytoplasm, and there was no obvious co-localization with the MDC dye (Figs 2D and S2D). This result indicated that MoMkk1 interacts with MoAtg4 in the cytoplasm.

To test whether MoAtg4 is also phosphorylated *in vivo*, proteins were extracted from Δ*Moatg4*/*MoATG4-GFP* and Δ*Momkk1*/*MoATG4-GFP* strains. Mn^2+^-Phos-tag SDS-PAGE showed that the phosphorylated MoAtg4 band was separated from the unphosphorylated one in the Δ*Moatg4*/*MoATG4-GFP* strain, while a similar pattern was observed in the Δ*Momkk1*/*MoATG4-GFP* strain (S2E Fig). Cell wall, ER, or nitrogen starvation stress all affect the function of MoMkk1 or MoAtg4 [5,16,22]. Thus, MoAtg4 phosphorylation under CFW, dithiothreitol (DTT), and nitrogen starvation minimal medium (MM-N) treatment was initially analyzed by phos-tag electrophoresis, and no difference was found under these conditions (S2F Fig). To further validate MoMkk1-dependent MoAtg4 phosphorylation, we purified the MoAtg4 protein from Δ*Moatg4*/*MoATG4-GFP* and Δ*Momkk1*/*MoATG4-GFP* strains and performed liquid chromatography-tandem mass spectrometry (LC-MS/MS) analysis with or without CFW. Data showed that serine 97 (S97), S276, and threonine 278 (T278) of MoAtg4 in Δ*Moatg4*/*MoATG4-GFP* treated with CFW, but not Δ*Momkk1*/*MoATG4-GFP* with CFW and the two strains without CFW treatment, were phosphorylated (Figs 2E and S2G). However, T101 was phosphorylated in both Δ*Moatg4*/*MoATG4-GFP* and Δ*Momkk1*/*MoATG4-GFP* strains, which was consistent with phos-tag electrophoresis results, suggesting that a complex MoAtg4 phosphorylation pattern (S2G Fig).

To validate S97, S276, and T278 are MoMkk1-dependent phosphorylation sites, we performed *in vitro* phosphorylation analysis using the nonphosphorylated protein His-MoAtg4^3A^ in which these residues were replaced with alanine (S97A, S276A, and T278A) and single-site phosphorylation mutations His-MoAtg4^S97A^, His-MoAtg4^S276A^, and His-MoAtg4^T278A^. Results showed that phosphorylation levels of MoAtg4^S97A^, MoAtg4^S276A^, and MoAtg4^T278A^ were significantly reduced compared with MoAtg4, while MoAtg4^3A^ did not react with MoMkk1 (Fig 2F), indicating that these residues of MoAtg4 are important for MoMkk1-dependent phosphorylation under cell wall stress, and kinases other than MoMkk1 might also phosphorylate MoAtg4 in *M*. *oryzae*.

### MoMkk1-dependent MoAtg4 phosphorylation plays a role in the development and virulence of *M*. *oryzae*

**Fig 3:**
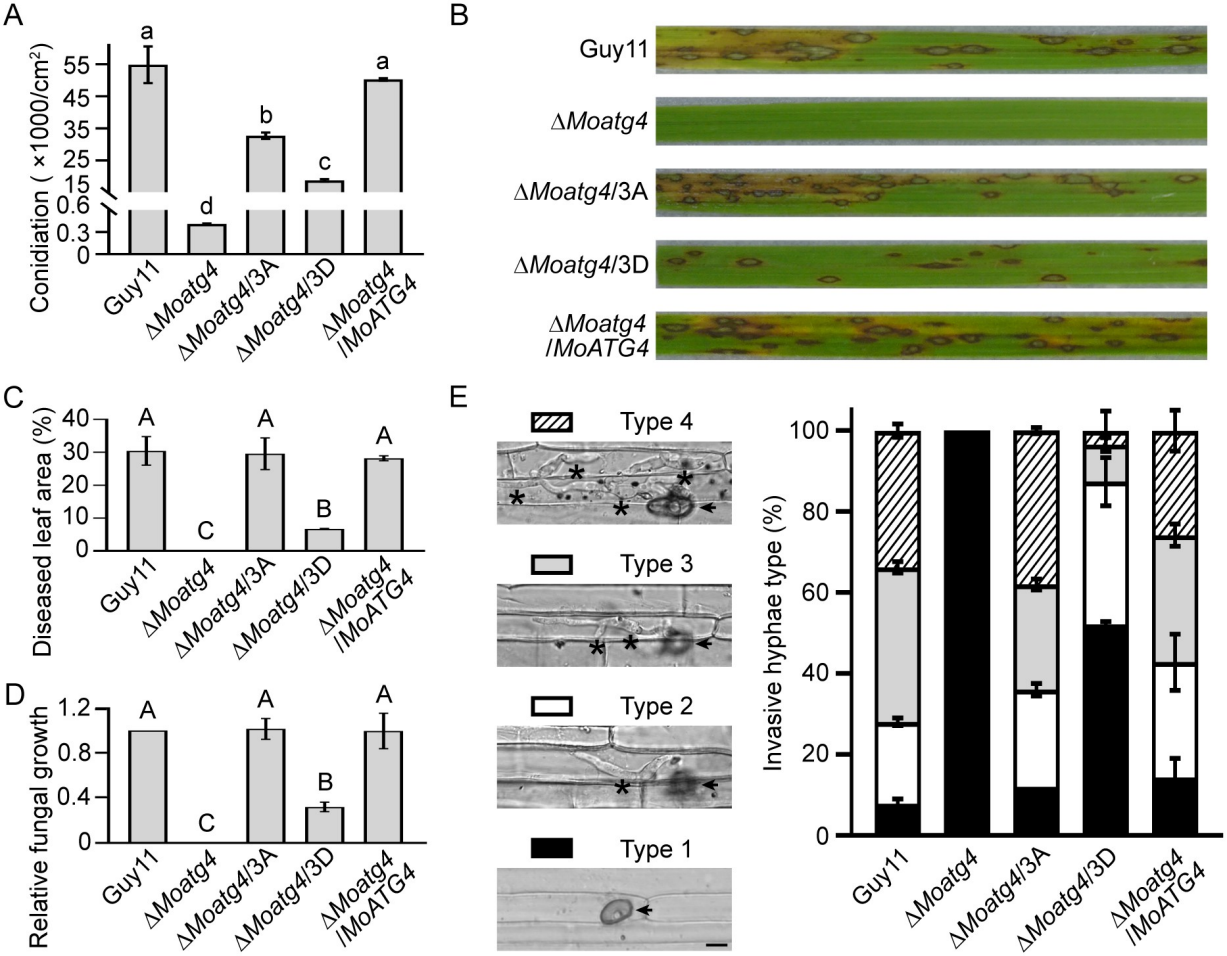
MoMkk1-dependent MoAtg4 phosphorylation functions in the development and virulence of *M*. *oryzae*. (A) Strains cultured on SDC (straw decoction and corn) medium at 28°C for 7 d in the dark, followed by 3 d of continuous illumination under fluorescent light for conidiation assay. (B) Conidial suspensions of Guy11, Δ*Moatg4*, Δ*Moatg4*/3A, Δ*Moatg4*/3D, and Δ*Moatg4*/*MoATG4* strains were sprayed onto 2-week-old rice seedlings (cultivar CO39) for virulence analysis. Diseased rice leaves were photographed at 7 days post inoculation (dpi). (C) Quantification of diseased leaf area as shown in (B) by Image J. (D) Severity of rice blasts was evaluated by quantitative PCR of *M*. *oryzae* genomic *28S rDNA* relative to rice genomic *RUBQ1* DNA as shown in (B). (E) Close observation and statistical analysis of the invasive hyphae (IH) growth in rice leaf sheath at 36 hours post inoculation (hpi). Statistical analysis of IH at 100 appressorium penetration sites by rating from type I to type IV (type 1, no penetration; type 2, a single IH with no branch; type 3, IH extended but was limited in one plant cell; type 4, IH extended to neighboring cells). The arrow points to appressorium, and the asterisk indicates IH. Scale bar: 5 μm. 3D: replaced S97, S276, and T278 with D to mimic phosphorylated form of MoAtg4. Different letters indicate statistically significant differences (Duncan’s new multiple range test, *p* < 0.05 or *p* < 0.01).

To investigate the biological function of MoMkk1-dependent MoAtg4 phosphorylation, we replaced S97, S276, and T278 residues with aspartic acid (D) to mimic phosphorylation, and introduced nonphosphorylated *MoATG4*^3A^-*GFP* and phosphorylation-mimic *MoATG4*^3D^-*GFP* vectors into the Δ*Moatg4* mutant, respectively. We found that Δ*Moatg4*/*MoATG4*^3A^ and Δ*Moatg4*/*MoATG4*^3D^ strains were defective in conidiation and appressorium formation. In addition, the phosphorylation-mimic strain showed more severe defects than nonphosphorylated ones (Figs 3A, S3A and S3B). The Δ*Moatg4*/*MoATG4*^3A^ strain showed a similar virulence as Guy11, in contrast to the attenuated one in the Δ*Moatg4*/*MoATG4*^3D^ strain (Fig 3B–3D). The invasive hyphae (IH) growth assay showed that about 30% of sites were type 4 in infection by Guy11, Δ*Moatg4*/*MoATG4*, and Δ*Moatg4*/*MoATG4*^3A^, whereas only approximately 5% were type 4 in infection by the Δ*Moatg4*/*MoATG4*^3D^ strain, and none by Δ*Moatg4* (Fig 3E). Collectively, these results indicated that MoMkk1-dependent MoAtg4 phosphorylation plays a role in the development and pathogenicity of *M*. *oryzae*.

To analyze the impact of cell wall stress on Δ*Moatg4*/*MoATG4*^3A^ and Δ*Moatg4*/*MoATG4*^3D^ strains, we investigated the growth, conidiation, and pathogenicity of the strains under CFW treatment. Although CFW inhibited 25% of the growth rate of Δ*Moatg4*/*MoATG4*^3A^ and 21% of Δ*Moatg4*/*MoATG4*^3D^ strains, it showed no difference compared to Guy11, and inhibitions of the strains’ virulence by CFW treatment were also similar. In addition, CFW treatment did not impact conidiation (S4A–S4C Fig). Since the CWI signaling pathway requires proper MAPK cascade phosphorylation and signal transduction [22], we examined the effect of MoMkk1-dependent MoAtg4 phosphorylation on CWI signaling through CFW staining. Chitin distribution of Δ*Moatg4*/*MoATG4*^3A^ and Δ*Moatg4*/*MoATG4*^3D^ strains is even and concentrated on the growing apices, and they also did not exhibit hyphal autolysis (S4D–S4F Fig). MoMps1 phosphorylation in Δ*Moatg4*/*MoATG4*^3A^ and Δ*Moatg4*/*MoATG4*^3D^ under CFW treatment was unaffected (S4G Fig), suggesting that MoMkk1-dependent MoAtg4 phosphorylation might not play a role in CWI signaling.

### MoMkk1-dependent MoAtg4 phosphorylation negatively controls autophagy by inhibiting MoAtg8 C-terminal cleavage

**Fig 4:**
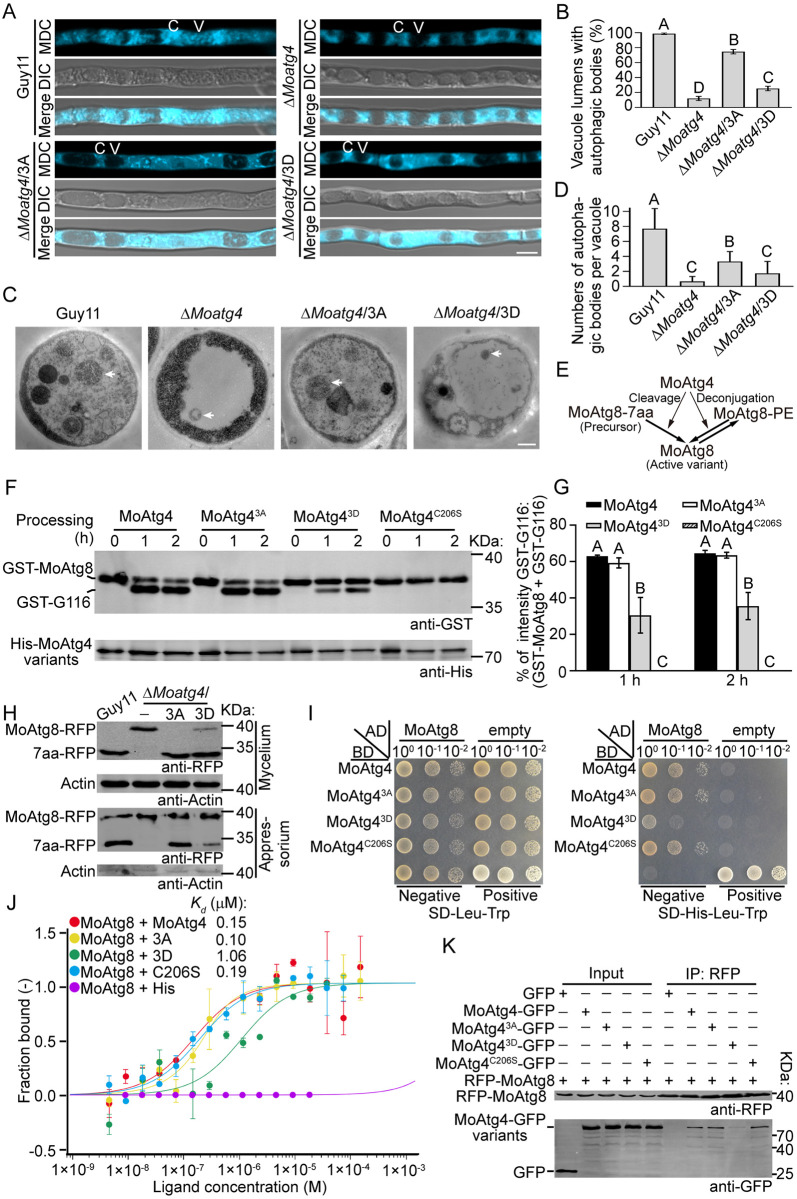
MoMkk1-dependent MoAtg4 phosphorylation negatively regulates autophagy by inhibiting MoAtg8 C-terminal cleavage. (A) Hyphae of Guy11, Δ*Moatg4*, Δ*Moatg4*/3A, and Δ*Moatg4*/3D strains were treated with MM-N for 5 h, and ABs were observed by confocal microscopy following MDC staining. C: cytoplasm. V: vacuole. Scale bar: 5 μm. (B) Quantification of vacuoles with AB as shown in (A). (C) Transmission electron microscopic (TEM) observation of AB in hyphae cultured in MM-N for 5 h. The arrow points to AB. Scale bar: 0.5 μm. (D) Quantification of AB as shown in (C). (E) Schematic diagram of MoAtg8 processing mediated by MoAtg4. MoAtg4 cleaves the C-terminus (7aa) of the MoAtg8 precursor to produce an active variant, then deconjugates MoAtg8-PE after autophagosome formation. (F) His-MoAtg4, His-MoAtg4^3A^, His-MoAtg4^3D^, and His-MoAtg4^C206S^ (protease-dead) fusion proteins were respectively mixed with GST-MoAtg8. After incubation at 37°C for 0, 1, or 2 h, *in vitro*, GST-MoAtg8 cleavage was analyzed by Western blot with anti-His and anti-GST antibodies. (G) Quantification of GST-MoAtg8 cleavage assays as shown in (E). (H) Extracted proteins from mycelia of Guy11, Δ*Moatg4*, Δ*Moatg4*/3A, and Δ*Moatg4*/3D strains expressing MoAtg8-RFP were treated with MM-N for 1 h and assayed for *in vivo* cleavage by Western blot using anti-RFP and anti-Actin antibodies. Conidia were inoculated to onion epidermis and proteins extracted at the appressorium developmental stage (8 hpi) and collected with anti-RFP agarose beads before analyzing MoAtg8 C-terminal cleavage. (I) pGBKT7 fused with *MoATG4*, *MoATG4*^3A^, *MoATG4*^3D^ or *MoATG4*^C206S^, and pGADT7 with *MoATG8* were co-introduced into the yeast AH109 strain and grew on SD-Leu-Trp, and the transformants were transferred to SD-His-Leu-Trp, or SD-Ade-His-Leu-Trp. Three colony solutions diluted to different levels (10^0^ to 10^−2^) of each protein pair were inoculated on the same plate for binding analysis. (J) 10 mM GST-MoAtg8 was labeled with RED-NHS and mixed with His-MoAtg4, MoAtg4^3A^, MoAtg4^3D^, and MoAtg4^C206S^ at various ranges of concentrations. Recombinant proteins were collected in NT standard capillaries for MST analysis that determined binding affinities. The empty His protein was used as a control. (K) Proteins were extracted from Δ*Moatg4*/*MoATG4*, Δ*Moatg4*/3A, Δ*Moatg4*/3D, and Δ*Moatg4*/C206S strains expressing RFP-MoAtg8, which were treated with MM-N for 1 h, and co-IP assays were performed. Western blot analysis was performed using anti-RFP and anti-GFP antibodies. Proteins from Δ*Moatg4* co-expressing GFP and RFP-MoAtg8 were used as a control. GST-G116: GST-MoAtg8^G116^. Different letters indicate statistically significant differences (Duncan’s new multiple range test, *p* < 0.01).

MoAtg4 was shown to regulate development and pathogenicity through autophagy function in *M*. *oryzae* [5,6]. To test whether MoMkk1-dependent MoAtg4 phosphorylation fulfills biological function by modulating autophagy, we performed MDC staining. About 99% of vacuoles contained AB in Guy11, but only 10% in the Δ*Moatg4* mutant. This autophagic defect was mostly restored by MoAtg4^3A^, in which 75% of the vacuoles contained AB, and was slightly restored in the Δ*Moatg4*/*MoATG4*^3D^ strain (25%) (Fig 4A and 4B). In addition, transmission electron microscopy (TEM) showed that AB numbers per vacuole were significantly reduced in the Δ*Moatg4*/*MoATG4*^3D^ strain (Fig 4C and 4D). We also assessed the effect of MoMkk1-dependent MoAtg4 phosphorylation on cell wall stress-induced autophagy, and found that autophagy was significantly decreased in the Δ*Moatg4*/*MoATG4*^3D^ strain (S5A and S5B Fig). These results demonstrated that MoMkk1-dependent MoAtg4 phosphorylation negatively regulates autophagy in *M*. *oryzae*.

The cysteine protease function of MoAtg4 responsible for MoAtg8 C-terminal cleavage and MoAtg8-PE deconjugation (Fig 4E) is required for autophagy [5]. We compared the MoAtg8 amino acid sequence with that of yeast Atg8 and found that MoAtg8 contains seven amino acid residues (7aa) following the active site glycine 116 (G116), instead of a single arginine residue in Atg8 (S5C Fig). To test whether MoAtg4 phosphorylation regulates the C-terminal cleavage of MoAtg8, the recombinant His-MoAtg4, His-MoAtg4^3A^, and His-MoAtg4^3D^ were incubated with GST-MoAtg8, respectively. The protease-dead MoAtg4^C206S^, which causes a serious autophagic defect, was used as a negative control [1,5]. The results showed that MoAtg4, but not MoAtg4^C206S^, cleaves MoAtg8, and the nonphosphorylated MoAtg4^3A^ exhibited a similar cleavage to MoAtg4, while the cleavage in the phosphorylation-mimic MoAtg4^3D^ was reduced (Fig 4F and 4G). Moreover, an *in vivo* cleavage assay was performed in Guy11, Δ*Moatg4*, Δ*Moatg4*/*MoATG4*^3A^, and Δ*Moatg4*/*MoATG4*^3D^ strains expressing MoAtg8-RFP, a MoAtg8 with a C-terminal RFP tag, under MM-N condition or CFW treatment. The bands of 7aa-RFP were detected in Guy11 and the Δ*Moatg4*/*MoATG4*^3A^ strains, while this cleavage was blocked in the Δ*Moatg4* mutant and drastically impaired in the Δ*Moatg4*/*MoATG4*^3D^ strain (Figs 4H and S5D).

*M*. *oryzae* infection is dependent on appressoria that penetrate plant epidermis and appressorial development requires autophagy [2,31,35,36]. We next observed the localization of MoAtg8-RFP in three-celled conidia and appressoria at the appressorium developmental stage. RFP fluorescence was mainly localized in vacuoles in conidium cells of Guy11 and Δ*Moatg4*/3A, while this localization pattern was not obvious in Δ*Moatg4* and Δ*Moatg4*/3D (S5E Fig). Western blot analysis further showed that MoAtg4^3D^ diminished ability to cleave MoAtg8 (Fig 4H). In contrast, MoAtg8 C-terminal cleavages were the same at the conidial stage in these strains (S5F Fig), suggesting that autophagy might not be induced. We also investigated MoAtg8 C-terminal cleavage in single-site phosphorylation mutations strains, Δ*Moatg4*/*MoATG4*^S97D^, Δ*Moatg4*/*MoATG4*^S276D^, and Δ*Moatg4*/*MoATG4*^T278D^, and found that C-terminal cleavage was all weakened but still stronger than Δ*Moatg4*/*MoATG4*^3D^ (S5D Fig), suggesting all individual phosphorylation residue is involved in MoAtg8 C-terminal cleavage. These results indicated that MoMkk1-dependent MoAtg4 phosphorylation inhibits the C-terminal cleavage of MoAtg8.

We next tested whether MoAtg4 phosphorylation affects its interaction with MoAtg8. The Y2H assay showed that, compared to MoAtg4, MoAtg4^3A^ and MoAtg4^C206S^ exhibit a similar binding, while MoAtg4^3D^ displays a significantly decreased interaction with MoAtg8 (Fig 4I). The microscale thermophoresis (MST) assay also showed that the interaction between MoAtg4^3D^ and MoAtg8 had a markedly higher dissociation constant (*K*_*d*_ = 1.06 μM) than that of MoAtg4 with MoAtg8 (*K*_*d*_ = 0.15 μM), MoAtg4^3A^ with MoAtg8 (*K*_*d*_ = 0.10 μM), and MoAtg4^C206S^ with MoAtg8 (*K*_*d*_ = 0.19 μM) (Fig 4J), which verified that MoAtg4^3D^ weakens its interaction with MoAtg8. Co-IP analysis further revealed an attenuated interaction between MoAtg4^3D^ and MoAtg8 under MM-N condition or CFW treatment (Figs 4K and S5G). These results suggested that MoMkk1-dependent phosphorylation disturbs the interaction of MoAtg4 with MoAtg8. We also investigated whether the phosphorylation affects the interaction between MoMkk1 and MoAtg4 by Y2H, and found that the interaction between MoAtg4^3A^ or MoAtg4^3D^ and MoMkk1 was the same as MoAtg4 with MoMkk1 (S5H Fig). Taken together, MoMkk1-dependent MoAtg4 phosphorylation inhibited the C-terminal cleavage of MoAtg8 to negatively control autophagy.

### MoAtg1-mediated MoAtg4 phosphorylation is involved in the development and pathogenicity of *M*. *oryzae*

Our previous analysis showed that MoAtg4 remains phosphorylated in the Δ*Momkk1* mutant (S2E and S2F Fig), suggesting a possibility of phosphorylation by other protein kinases. Since the yeast and human Atg1/Ulk1 phosphorylate Atg4/Atg4B [1,37], we tested whether MoAtg1 also phosphorylates MoAtg4. The interaction between MoAtg1 and MoAtg4 was first observed at PAS (S6A and S6B Fig). The *in vitro* phosphorylation assay showed MoAtg1 phosphorylates MoAtg4 (S6C Fig). Further LC-MS/MS data revealed that the phosphorylation of MoAtg4 at S67 was detected in Guy11, but not in the Δ*Moatg1* mutant, under nitrogen starvation, Guy11 and Δ*Moatg1* without starvation (S6D Fig). Although not covered in repeated LC-MS/MS data, S364, a conserved residue previously reported to be phosphorylated by Atg1 in *S*. *cerevisiae*, was mutated to evaluate the function in MoAtg1-mediated phosphorylation reaction (S6E Fig). *In vitro* phosphorylation analysis showed that phospho-fluorescence was partially reduced in MoAtg4^S67A^ and MoAtg4^S364A^, while MoAtg4^2A^ containing mutations of both S67A and S364A showed more significantly reduced phospho-fluorescence (S6C Fig), indicating that S67 and S364 residues are important for MoAtg4 phosphorylation by MoAtg1.

To investigate the role of MoAtg1-mediated MoAtg4 phosphorylation in *M*. *oryzae*, we generated nonphosphorylated Δ*Moatg4*/*MoATG4*^2A^ and phosphorylation-mimic Δ*Moatg4*/*MoATG4*^2D^ strains, and single-site phosphorylation mutations strains, including Δ*Moatg4*/*MoATG4*^S67A^, Δ*Moatg4*/*MoATG4*^S364A^, Δ*Moatg4*/*MoATG4*^S67D^, and Δ*Moatg4*/*MoATG4*^S364D^. Compared with Guy11, Δ*Moatg4*/*MoATG4*^2A^ and Δ*Moatg4*/*MoATG4*^2D^ strains showed defects in conidiation and appressorium formation. In addition, MoAtg4^2D^ caused more severe defects than MoAtg4^2A^, and MoAtg4^2A^ resulted in more severe defects than MoAtg4^S67A^ or MoAtg4^S364A^, and similarly, MoAtg4^2D^ produced more severe defects than MoAtg4^S67D^ or MoAtg4^S364D^ (S3C, S3D and S6F Figs). Moreover, Δ*Moatg4*/*MoATG4*^2D^ exhibited reduced pathogenicity and more severe than Δ*Moatg4*/*MoATG4*^S67D^ and Δ*Moatg4*/*MoATG4*^S364D^, while the Δ*Moatg4*/*MoATG4*^S67A^, Δ*Moatg4*/*MoATG4*^S364A^, and Δ*Moatg4*/*MoATG4*^2A^ strains were as virulent as Guy11 (S6G and S6H Fig). We therefore concluded that MoAtg1-mediated MoAtg4 phosphorylation is also involved in appressorium formation and pathogenicity of *M*. *oryzae*.

### MoAtg1-mediated MoAtg4 phosphorylation negatively regulates autophagy by inhibiting deconjugation of MoAtg8-PE

Since both MoAtg1 and MoAtg4 are essential for autophagy, we next investigated the effect of MoAtg1-mediated MoAtg4 phosphorylation on autophagy in *M*. *oryzae*. MDC staining showed that the number of vacuoles with AB was nearly 100% in Guy11, but reduced to 77% in Δ*Moatg4*/*MoATG4*^2A^ and 28% in Δ*Moatg4*/*MoATG4*^2D^ strains, respectively (S7A and S7B Fig). Consistently, TEM showed defective autophagy in the aberrantly phosphorylated strains (S7C and S7D Fig). These results indicated that MoAtg1-mediated MoAtg4 phosphorylation is critical for autophagy.

In yeast, Atg4 is responsible for Atg8-PE deconjugation and Atg8 recycling to promote the fusion of autophagosomes with vacuoles and flowing autophagosomes formation, which is inhibited by Atg1-mediated Atg4 phosphorylation [1,38]. To assay whether the phosphorylation of MoAtg4 controls the deconjugation of MoAtg8-PE, we expressed MoAtg8^G116^ with an N-terminal RFP tag (RFP-MoAtg8^G116^), which could conjugate to PE without the initial step of MoAtg4-mediated cleavage [1,38], in the Δ*Moatg4*/*MoATG4*^2A^ and Δ*Moatg4*/*MoATG4*^2D^ strains. As indicated by vacuolar fluorescent dye CMAC [1,39], RFP signals were localized in the vacuoles of Guy11 and Δ*Moatg4*/*MoATG4*^2A^ strains but were restricted in the cytoplasm and the edge of vacuoles in Δ*Moatg4* and Δ*Moatg4*/*MoATG4*^2D^ strains (S7E Fig). Blocked deconjugation of MoAtg8-PE was also observed at the appressorium maturing stage in Δ*Moatg4* and Δ*Moatg4*/*MoATG4*^2D^ strains. Western blotting analysis showed that protein levels of free RFP were lower in Δ*Moatg4* and Δ*Moatg4*/*MoATG4*^2D^ (S7F Fig). These findings suggest that the phosphorylation of MoAtg4 by MoAtg1 inhibits deconjugation of MoAtg8-PE.

To understand the underlying mechanism, we examined whether MoAtg4 phosphorylation by MoAtg1 affects its interaction with MoAtg8. Y2H results showed that the phosphorylation-mimic MoAtg4^2D^, but not the nonphosphorylated variant MoAtg4^2A^, significantly reduced the affinity with MoAtg8 (S7G Fig). Co-IP analysis was also performed with proteins extracted from strains expressing RFP-MoAtg8^G116^, and a weak interaction was detected between MoAtg4^2D^ and MoAtg8 (S7H Fig). Interestingly, vacuole-localized RFP signals were observed in both MoMkk1-dependent nonphosphorylated Δ*Moatg4*/*MoATG4*^3A^ and phosphorylation-mimic Δ*Moatg4*/*MoATG4*^3D^ strains, which expressed RFP-MoAtg8^G116^ (S8A Fig), while this localization was not observed in Δ*Momck1*, Δ*Momkk1*, and Δ*Momps1* (S8B Fig), suggesting that MoMck1, MoMkk1, and MoMps1, but not MoMkk1-dependent MoAtg4 phosphorylation, is involved in the deconjugation of MoAtg8-PE. Overall, MoAtg1-mediated MoAtg4 phosphorylation negatively regulates autophagy by inhibiting deconjugation of MoAtg8-PE at PAS.

### MoAtg4 phosphorylation mediated by MoMkk1 and MoAtg1 orchestrates autophagy and pathogenicity of *M*. *oryzae*

As MoMkk1 and MoAtg1 phosphorylate MoAtg4 at different amino acid residues to regulate the autophagy, development, and pathogenicity of *M*. *oryzae*, we examined the synergistic effect of the two phosphorylation events. All phosphorylation sites mediated by both kinases were mutated to A or D to construct the nonphosphorylated MoAtg4^5A^ or phosphorylation-mimic MoAtg4^5D^ variant, respectively. Interestingly, MoAtg4^5A^ rescued defects of the Δ*Moatg4* mutant in conidiation, appressorium formation, and pathogenicity, similar to MoAtg4^3A^ and MoAtg4^2A^, while MoAtg4^5D^ did not show any restoring effect (S3A, S3B and S9A–S9E Figs). In short, MoMkk1 and MoAtg1 phosphorylate MoAtg4 to collectively regulate the development and pathogenicity of *M*. *oryzae*.

**Fig 5:**
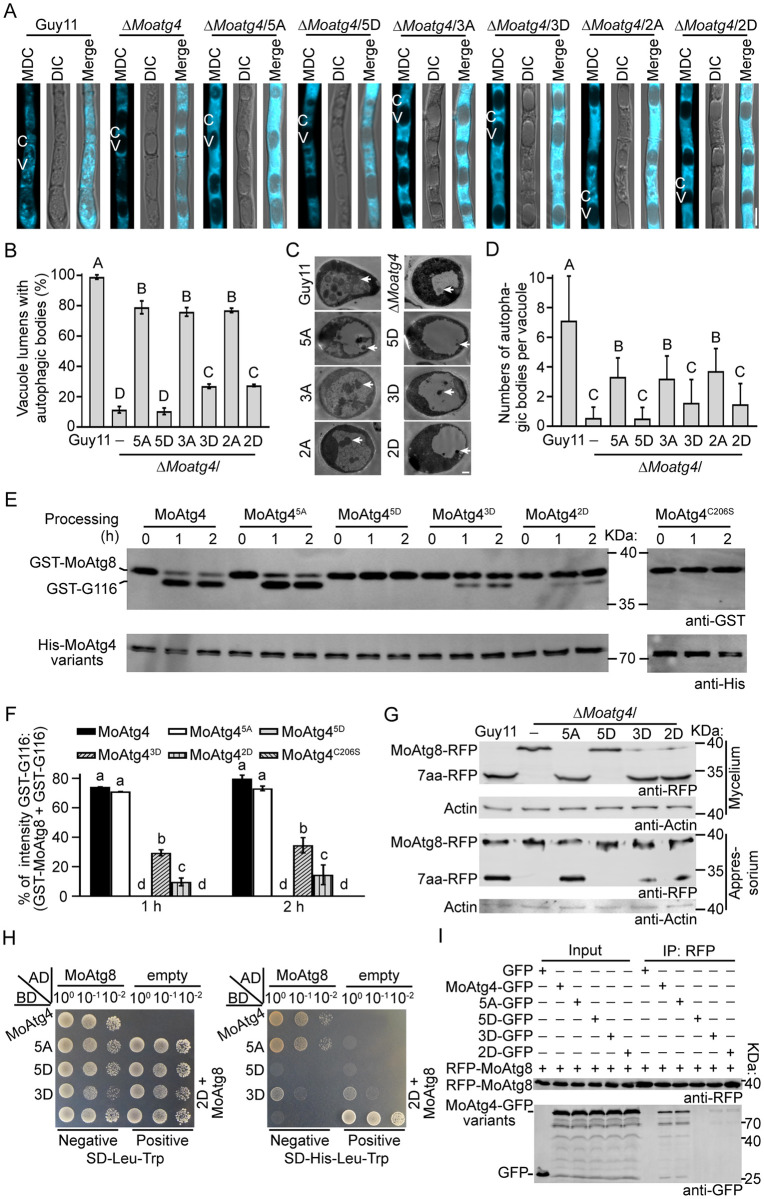
MoAtg4 phosphorylation mediated by MoMkk1 and MoAtg1 orchestrates autophagy. (A) AB observation after MDC staining. C: cytoplasm. V: vacuole. Scale bar: 5 μm. (B) Quantification of vacuoles with AB as shown in (A). (C) AB observation using TEM. The arrow points to AB. Scale bar: 0.5 μm. (D) Quantification of AB as shown in (C). (E) Cleavage assay of GST-MoAtg8 *in vitro*. (F) Quantification of GST-MoAtg8 cleavage assay as shown in (E). (G) *In vivo* cleavage assay of MoAtg8-RFP at the mycelium (nitrogen starvation for 1 h) and appressorium stages. (H) The binding ability analysis of MoAtg4^5A^ and MoAtg4^5D^ with MoAtg8 by Y2H. (I) Co-IP assay to analyze the interaction between MoAtg4^5A^ or MoAtg4^5D^ and MoAtg8. 5A/5D: All MoAtg4 phosphorylation sites (S97, S276, T278, S67, and S364) mediated by MoMkk1 and MoAtg1 were replaced by A/D to mimic nonphosphorylated/phosphorylated form of MoAtg4. GST-G116: GST-MoAtg8^G116^. Different letters indicate statistically significant differences (Duncan’s new multiple range test, *p* < 0.05 or *p* < 0.01).

We next analyzed autophagic levels in Δ*Moatg4*/*MoATG4*^5A^ and Δ*Moatg4*/*MoATG4*^5D^ strains. Like Δ*Moatg4*, the Δ*Moatg4*/*MoATG4*^5D^ strain only had 10% vacuoles containing AB, with the AB number per vacuole significantly reduced compared to Guy11 (Fig 5A–5D). The *in vitro* and *in vivo* C-terminal cleavage assays of MoAtg8 showed that MoAtg4^5D^ could not cleave MoAtg8, similar to MoAtg4^C206S^, and that the C-terminal cleavage did not occur at the mycelial or appressorial stage in the Δ*Moatg4*/*MoATG4*^5D^ strain (Fig 5E–5G). Interestingly, MoAtg4^5D^ could not interact with MoAtg8 (Fig 5H and 5I). These results indicated that MoAtg4 phosphorylation mediated by MoMkk1 and MoAtg1 collaborates to negatively regulate autophagy of *M*. *oryzae*.

In order to explore the relationship between MoMkk1- and MoAtg1-dependent MoAtg4 phosphorylation, we performed *in vitro* phosphorylation reactions of MoMkk1 with MoAtg4^2D^ and MoAtg1 with MoAtg4^3D^. Interestingly, phosphorylation of MoMkk1 with MoAtg4^2D^ and MoAtg1 with MoAtg4^3D^ were significantly weaker than them with MoAtg4 (S9F Fig), suggesting an antagonistic relationship between MoMkk1- and MoAtg1-dependent MoAtg4 phosphorylation. MoMkk1^S115D^, the phosphorylated form by MoAtg1 [16], was also tested for phosphorylation with MoAtg4, and results showed that the phosphorylation level was similar, compared to MoMkk1 with MoAtg4 (S9G Fig), indicating that the action of MoAtg1 on MoMkk1 does not affect MoMkk1-dependent MoAtg4 phosphorylation. MoMkk1 phosphorylation in Δ*Moatg4*/*MoATG4*^3A^ and Δ*Moatg4*/*MoATG4*^3D^ treated with DTT showed no difference compared with Δ*Momkk1*/*MoMKK1*-*RFP* (S9H Fig), suggesting that MoMkk1-dependent MoAtg4 phosphorylation does not influence MoMkk1 phosphorylation of MoAtg1. In addition, MoAtg8 C-terminal cleavages in Δ*Momkk1*/*MoMkk1*^S115A^ and Δ*Momkk1*/*MoMkk1*^S115D^ were as the same as Guy11 with CFW treatment (S9I Fig), indicating that MoMkk1 phosphorylation by MoAtg1 does not affect the cleavage activity of MoAtg4. Interestingly, MoAtg8 C-terminal cleavage in Δ*Momck1* and Δ*Momkk1* under CFW treatment was stronger, while the cleavage in Δ*Momps1* was the same as Guy11 (S9I Fig). We inferred that the cleavage in Δ*Momps1* was inhibited by MoMkk1-dependent MoAtg4 phosphorylation, but the loss of MoAtg4 phosphorylation resulted in stronger MoAtg8 cleavage in Δ*Momck1* and Δ*Momkk1*. Taken together, MoAtg4 phosphorylation mediated by MoMkk1 and MoAtg1 collectively govern the autophagy and pathogenicity in *M*. *oryzae*.

## Discussion

In eukaryotic cells, CWI signals are amplified by the MAPK pathway to maintain cell wall integrity, whereas autophagy is activated to degrade intracellular components for cellular homeostasis [15,40]. Recently, ATG proteins in *M*. *oryzae* were found to enhance CWI signaling under ER stress generated by abnormal protein synthesis [16]. In turn, the components of the CWI signaling pathway also affect autophagy. In the budding yeast, Slt2 regulates selective autophagy, but not nonselective autophagy, while CWI pathway components are important for nonselective autophagy in *M*. *oryzae* [16,26–28]. However, it is unknown whether CWI kinases regulate autophagy through ATG protein phosphorylation. Here, we found that MoMkk1 phosphorylates MoAtg4 to maintain proper autophagic levels under cell wall stress. It is worth noting that autophagy is a double-edged sword since either too low or too high levels are not conducive to normal survival and development [41,42]. Although MoMkk1-dependent MoAtg4 phosphorylation negatively regulates autophagy, previous studies suggested that Pak1 (p21 [Rac1] activated kinase) phosphorylates Atg5 to promote human cell autophagy by strengthening its interaction with Atg16L [43]; therefore, the CWI signaling pathway may enhance autophagy through other factors under cell wall stress. Besides, our results indicate that cell wall stress does not induce ER-phagy, which is different from the CWI signaling pathway reported in yeast, highlighting a distinctive aspect of our investigation.

Our results further showed that the C-terminal cleavage of MoAtg8 is stronger in Δ*Momck1* and Δ*Momkk1*, but remains unchanged in Δ*Momps1* under cell wall stress. This is probably due to the absence of MoMkk1-dependent MoAtg4 phosphorylation, either through the blockage of cell wall stress transmission or deletion of kinase MoMkk1 in Δ*Momck1* and Δ*Momkk1* [22]. In Δ*Momps1*, however, the normal level of MoAtg4 phosphorylation inhibits MoAtg8 cleavage and subsequent autophagy. Together, MoMkk1-dependent MoAtg4 phosphorylation mediates the coordination between autophagy and the CWI pathway in *M*. *oryzae*.

During *M*. *oryzae* infection of host plant, the fungus faces cell wall and nitrogen starvation stresses caused by cell wall remodeling and nutrient deficiency [6,14,31]. How does the fungus overcome or respond to these conditions? Our studies indicated that MoMkk1 phosphorylates MoAtg4 under cell wall stress and that MoAtg1 phosphorylates MoAtg4 under nitrogen starvation stress, which are involved in autophagy regulation, thus affecting appressorium-mediated infection of host and pathogenicity. Therefore, our research demonstrated the importance of CWI kinase-dependent ATG protein phosphorylation in *M*. *oryzae*.

In yeast, the C-terminal cleavage of Atg8 and deconjugation of Atg8-PE mediated by Atg4 are important for autophagosome formation and autophagy [38,44]. Atg1 phosphorylates Atg4 at PAS to enhance autophagy by protecting the Atg8-PE pool [1]. We asked whether organisms employ Atg4 phosphorylation to accurately distinguish the two different processing of Atg8 and control autophagy. In this study, we showed that MoAtg1 phosphorylates MoAtg4 at S67 and S364 (the latter is same as yeast Atg4) to inhibit deconjugation of MoAtg8-PE similar to yeast, while MoMkk1-mediated MoAtg4 phosphorylation is responsible for the C-terminal cleavage of MoAtg8 in the cytoplasm. Our results suggested that the phosphorylation of MoAtg4 by different kinases regulates MoAtg8 processing in a delicate spatiotemporal manner. It has been reported that mammalian sterile20-like kinase Mst4, with the exception of Ulk1, phosphorylates Atg4B to promote autophagy [45]. *M*. *oryzae* might be more similar to mammals than yeast in that there are additional MoAtg4 regulatory mechanisms. Furthermore, MoMkk1- and MoAtg1-mediated MoAtg4 phosphorylation inhibits the interaction of MoAtg4-MoAtg8. Interestingly, MoAtg4^C206S^ cannot cleave MoAtg8, but it interacts with MoAtg8 in a manner similar to MoAtg4. This suggests the phosphorylation of MoAtg4 may directly influence cleavage and modulate cleavage through altering MoAtg4-MoAtg8 interaction, ultimately regulating autophagy.

The deconjugation of Atg8-PE enhances autophagy by promoting the fusion of autophagosomes with vacuoles and also releasing and recycling Atg8 into the cytosol [12, 46]. Compared with *de novo* protein synthesis, recycling is a more efficient process. In *M*. *oryzae*, MoPtps protein phosphatases regulate MoOsm1, a Hog1 homolog, recycling into the cytoplasm through dephosphorylation to enable a rapid response to the external stress [47]. Likewise, the late endosomes circulate the G-protein regulator MoRgs7 and the G-protein Gα subunit MoMagA to the plasma membrane to perceive the hydrophobic surface and promote cAMP signal transduction [48]. Our studies revealed a similar recycling mechanism of MoAtg8 existed in *M*. *oryzae*: MoAtg4 deconjugates and recycles MoAtg8 from the MoAtg8-PE anchor localized on the autophagosomal membrane to the cytoplasm. Of note, MoAtg1-mediated MoAtg4 phosphorylation may inhibit this process on immature autophagosomes. Although MoAtg8 recycling increases the MoAtg8 pool in the cytosol, which is available for subsequent autophagosome formation, earlier MoAtg8 recycling may prevent completion of current autophagosomes. Therefore, the inhibition of MoAtg8 deconjugation by MoAtg1-mediated phosphorylation may balance the MoAtg8 demand for autophagosome formation and recycling to modulate development and virulence.

One acceptor protein can be phosphorylated by multiple kinases is an efficient but complex regulation mechanism. MoMkk1 is a core detector that can be phosphorylated by MoMck1 and MoSep1, and as well as MoAtg1 under cell wall or ER stress [14,16]. The mammalian Ulk1 is phosphorylated by the AMP-activated protein kinase (AMPK) and mammalian target of rapamycin (mTOR) to promote or inhibit its activity under glucose starvation or nutrient sufficiency conditions, respectively [49]. How do cells sort out these phosphorylation relationships? Our study showed that MoMkk1- and MoAtg1-dependent MoAtg4 phosphorylation are contrary to each other, though it is difficult to quantify phosphorylation under cell wall and nutrition stress *in vivo* due to its complexity, as MoAtg4 phosphorylation is still detectable in the Δ*Moatg1*Δ*Momkk1* double deletion mutant (S2E Fig). MoAtg4 is localized in both the cytoplasm and at PAS, and MoMkk1 is mainly located in the cytoplasm. It has been reported that MoAtg1 is perceptible in the cytoplasm of *M*. *oryzae* [4], and yeast Atg1 gathers to PAS during autophagy [50, 51], which probably causes more binding of MoAtg1 but not MoMkk1 and phosphorylates MoAtg4 at PAS. On the other hand, the antagonistic relationship of the two MoAtg4 phosphorylation events guarantees their collaborative but differential function.

**Fig 6:**
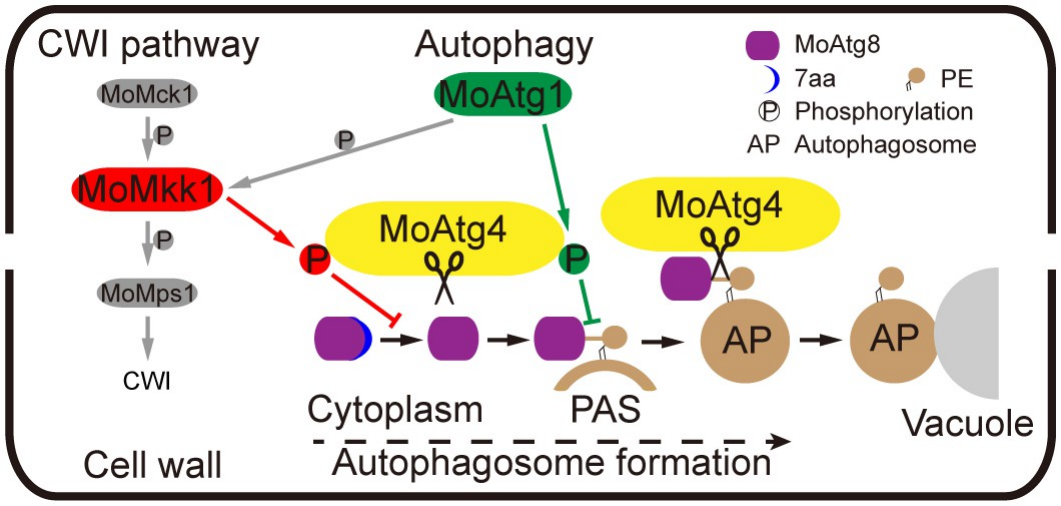
A model of autophagy and CWI pathways coordinating development and pathogenicity through MoAtg4 phosphorylation. MoAtg4 cleaves the MoAtg8 C-terminus (7aa) to activate the conjunction of MoAtg8 with phosphatidylethanolamine (PE) of PAS. Following autophagosome formation, MoAtg4 deconjugates MoAtg8 from PE anchors to promote the fusion of autophagosomes with vacuoles. The CWI kinase MoMkk1 phosphorylates MoAtg4 in the cytoplasm to attenuate the C-terminal cleavage of MoAtg8. On the other hand, The ATG protein kinase MoAtg1 phosphorylates MoAtg4 at PAS to inhibit the deconjugation of MoAtg8-PE. Two MoAtg4 phosphorylation events collectively maintain proper autophagic levels to govern the development and pathogenicity of *M*. *oryzae*.

In summary, MoAtg4 phosphorylation events mediated by MoAtg1 and MoMkk1 revealed novel crosstalk between autophagy and CWI signaling. MoAtg1 targets and phosphorylates MoAtg4 at PAS to inhibit deconjugation of MoAtg8-PE, whereas MoMkk1 phosphorylates MoAtg4 in the cytoplasm to reduce the C-terminal cleavage of MoAtg8. Together, they coordinate proper autophagic levels for regulating the development and pathogenicity of *M*. *oryzae* (Fig 6).

## Materials and methods

### Strains and culture conditions

*M*. *oryzae* strain Guy11 was used as the wild-type strain in this study. Fusion constructs containing the MoAtg4 promoter, MoAtg4 (or sites-mutation variants) coding region, fragments of GFP, and bleomycin were transferred to Δ*Moatg4* mutant to obtain complemented and phosphorylation site-mutations strains. All strains were cultured on CM at 28°C in the dark. Mycelia blocks were cut from plates and cultivated in liquid CM for 2 d at 28°C, and mycelia were harvested for DNA, RNA, and protein extraction. For the conidiation assay, strains were cultured on an SDC medium in the dark at 28°C for 7 d, followed by 3 d illumination under fluorescent light before counting [52, 53]. Conidia were adjusted to a concentration of 1 × 10^5^/ml, and 30 μl of conidial solution was inoculated to hydrophobic slides. Conidial germination and appressorium formation were observed using a microscope following incubation in the dark for 2, 6, 10, and 24 h, respectively.

### Virulence assay

For the virulence assay, conidia were harvested and resuspended to 5 × 10^4^/ml with 0.2% (w:v) gelatin. 5 ml of solution was sprayed on two-week-old rice seedlings (*Oryza sativa* cultivar CO39). The inoculated plants were incubated in the dark at 28°C and 90% humidity for 24 h, then transferred to 12 h light-dark environment for 6 d to assess leaf symptoms, which was quantified by measuring lesion area and quantitative PCR of *M*. *oryzae* genomic *28S rDNA* relative to rice genomic *RUBQ1* DNA [54]. For IH growth analysis, 3-week-old rice sheaths were injected with conidial suspension (1 × 10^5^ spores/ml) and cultured in the dark at 28°C with high humidity for 36 h [55].

### Yeast-two-hybrid (Y2H) assay

The coding sequences of corresponding genes were amplified and cloned into pGBKT7 or pGADT7 vectors, which were then introduced into the yeast AH109 strain after verification. Transformants grown on synthetic dextrose medium lacking tryptophan and leucine (SD-Leu-Trp) and were transferred to the medium lacking tryptophan, leucine, and histidine (SD-His-Leu-Trp) or tryptophan, leucine, histidine, and adenine (SD-Ade-His-Leu-Trp) [47].

### GST pull-down assay

GST, GST-MoMkk1, and His-MoAtg4 were expressed in *Escherichia coli* BL21 cells. Cells were collected and washed 3 times with 1× PBS, then resuspended and lysed with a sonicator. After centrifugation with 13,000 g for 10 min, the GST or GST-MoMkk1 supernatant was mixed with 30 μl of glutathionesepharose beads (GE Healthcare, 10265165), which were washed 3 times using GST washing buffer (250 mM NaCl, 20 mM Tris, 2 mM EDTA, 2 mM EGTA, pH 7.5), then incubated at 4°C for 2 h. The supernatant was discarded after centrifugation (13,000 g, 10 min) and the GST- or GST-MoMkk1-bound to GST beads were incubated with His-MoAtg4 protein solution for another 4 h. The beads were washed 5 times with GST washing buffer. Proteins were eluted with GST elution buffer (10 mM reduced glutathione in GST washing buffer, pH 8.0), then analyzed by Western blot analysis with anti-His (Abmart, M20001) and anti-GST (Abmart, M20007) antibodies, respectively.

### Co-immunoprecipitation (co-IP) assay

MoMkk1-GFP and MoAtg4-Flag were co-expressed in Guy11. Total mycelial proteins were extracted and incubated with anti-GFP beads (KT HEALTH, KTSM1301) at 4°C for 2 h, which were pre-washed 3 times with a GFP washing buffer (150 mM NaCl, 10 mM Tris-Cl, 0.5 mM EDTA, pH 7.5). Proteins bound to beads were washed 5 times with GFP washing buffer and eluted with 200 mM glycine (pH 2.5), then neutralized with 1 M Tris (pH 10.4). Input and IP proteins were detected by Western blot using anti-GFP (Engibody Biotechnology, AT0028) and anti-Flag antibodies (Engibody Biotechnology, AT0022), respectively [56].

### Bimolecular fluorescence complementation (BiFC) assay

nYFP-MoMkk1 and MoAtg4-cYFP were expressed in Guy11. Transformants were isolated from plates containing 150 μg/ml hygromycin and 200 μg/ml zeocin and confirmed by fluorescent microscopy. For the BiFC assay, YFP signals were examined with confocal microscopy during different stages of the transformants. Strains expressing empty nYFP with MoAtg4-cYFP and nYFP-MoMkk1 with empty cYFP were used as controls.

### *In vivo* phosphorylation analysis and phosphorylation site identification

To analyze the phosphorylation of MoAtg4 in *M*. *oryzae*, the *MoATG4-GFP* fusion construct was transferred into Δ*Moatg4*, Δ*Momkk1*, Δ*Moatg1*, and Δ*Moatg1*Δ*Momkk1* mutants, respectively. Mycelia powder was added into 1 ml of lysis buffer (50 mM Tris, 150 mM NaCl, 1 mM EDTA, 1% (v/v) Triton X-100, pH 7.4) with or without 10 μl of phosphatase inhibitor cocktail 3 (Sigma-Aldrich, P0044). Extracted proteins were then purified with anti-GFP beads. Alkaline phosphatase (Sigma-Aldrich, P6774) at a final concentration of 2.5 U/ml was added to half of the sample without cocktail 3 and incubated with 1 mM MgCl_2_ for 2 h at 37°C. These protein samples were separated on 8% SDS-PAGE gel containing 20 μM phos-tag (NARD institute Limited company, 18D01) and 100 μM MnCl_2_. After electrophoresis at 80 V for 5–8 h, the gel was equilibrated firstly in transfer buffer containing 5 mM EDTA twice, furthermore equilibrated another twice in transfer buffer without EDTA, each time for 20 min. Proteins were transferred from the gel to PVDF membrane at a constant pressure of 80 V for 36–48 h on ice, and then analyzed for phosphorylation by Western blot analysis using an anti-GFP antibody.

To identify MoMkk1- or MoAtg1-dependent MoAtg4 phosphorylation sites, mycelia proteins were extracted from Δ*Moatg4*/*MoATG4-GFP* and Δ*Momkk1*/*MoATG4-GFP* strains treated with 1 mg/ml CFW for 5 h or without, and Δ*Moatg4*/*MoATG4-GFP* and Δ*Moatg1*/*MoATG4-GFP* strains induced with MM-N for 5 h or without. After purification by anti-GFP beads, protein samples were separated on 12% SDS-PAGE and stained by Coomassie brilliant blue. MoAtg4-GFP bands were sliced, and phosphorylation sites were identified (Beijing Protein Innovation) by LC-MS/MS. Two independent experiments were used for phosphorylation site identification.

Phospho-p44/42 MAPK antibody (Cell Signaling Technology, 4370) was used to detect phosphorylated MoMps1 and p44/42 MAPK antibody (Cell Signaling Technology, 9102) was used as control.

### *In vitro* phosphorylation analysis

A rapid and cost-effective fluorescence detection in tube (FDIT) method using phosphoprotein gel stain was used for *in vitro* phosphorylation analysis [33,34,57]. Proteins GST-MoMkk1, His-MoAtg4, His-MoAtg3, GST-MoAtg5, and His-MoAtg16 were expressed in *E*. *coli* BL21 cells and purified using anti-GST or anti-His beads. The following reagents were placed in a 1.5 ml centrifuge tube: 0.2 μg kinase GST-MoMkk1, 2 μg substrate His-MoAtg4, His-MoAtg3, GST-MoAtg5, or His-MoAtg16 with ATP at a final concentration of 50 μM (Sigma-Aldrich, FLAAS), and sufficient kinase reaction buffer (100 mM PBS, 10 mM MgCl_2_, 1 mM ascorbic acid, pH 7.5) to 100 μl volume for phosphorylation reaction at room temperature for 1 h, then 10-fold of cold acetone was added to stop the reaction. Phosphorylation protein was stained by Pro-Q Diamond Phosphorylation Gel Stain (Thermo Fisher Scientific, P33301), the widely used phosphor-protein gel-staining fluorescence dye, in the dark at room temperature for 1 h. Proteins were pelleted with cold acetone and washed twice with 0.5 ml of cold acetone. The protein pellet then was dissolved in 200 μl of ddH_2_O after drying and measured phospho-fluorescence at 590 nm (excited at 530 nm) using a Cytation3 microplate reader (Biotek, Winooski, VT, USA). Groups without kinase or substrate proteins were set up as controls [14].

### Microscopic observation

Strains were cultured in liquid CM and transferred to MM-N or CM containing 1 mg/ml CFW (both adding 2 mM PMSF) [2] for 5 h. 50 μM MDC (Sigma-Aldrich, D4008) was used to stain AB at dark for 20–30 min and the observation was performed using a Zeiss LSM710 microscope. For the analysis of chitin distribution, hyphae cultured in CM were stained with CFW at a final concentration of 10 μg/ml in the dark for 5 min, being washed with distilled water before observation.

### *In vitro* and *in vivo* MoAtg8 cleavage assays

GST-MoAtg8, His-MoAtg4, His-MoAtg4^3A^, His-MoAtg4^3D^, and His-MoAtg4^C206S^ proteins were expressed in *E*. *coli* BL21 cells. 1 μg His-MoAtg4, His-MoAtg4^3A^, His-MoAtg4^3D^ or His-MoAtg4^C206S^ were mixed with 3 μg GST-MoAtg8 in a final volume of 1 ml. Following incubation at 37°C for 0, 1, or 2 h, the loading buffer was used to stop the reaction. The *in vitro* cleavage of GST-MoAtg8 was analyzed by Western blot with anti-His and anti-GST antibodies.

For *in vivo* MoAtg8 cleavage assay, proteins were extracted from Guy11, Δ*Moatg4*, Δ*Moatg4*/*MoATG4*^3A^, and Δ*Moatg4*/*MoATG4*^3D^ strains expressing the MoAtg8-RFP (growing in CM for 36 h and then transferred to MM-N for 1 h) and analyzed by Western blot with anti-RFP (Chromotek, 6g6-150) and anti-Actin (Nanjing Huading Biotechnology, 10011) antibodies. For MoAtg8 cleavage assay during appressorium formation, 5 ml of conidia (1 × 10^6^/ml) were inoculated to onion epidermis, then extracted proteins at 8 hpi, which were collected with anti-RFP agarose beads (MBL Beijing Biotech, M165-8) to perform Western blot.

### Microscale thermophoresis (MST) analysis

Binding affinities of MoAtg4, MoAtg4^3A^, MoAtg4^3D^, and MoAtg4^C206S^ with MoAtg8 were measured by MST in a Monolith NT.Label Free instrument (Nano Temper Technologies, GMBH). 10 mM GST-MoAtg8 was labeled with RED-NHS and mixed with a range of concentrations of His-MoAtg4, His-MoAtg4^3A^, His-MoAtg4^3D^, and His-MoAtg4^C206S^ (1:1, v/v). An empty His protein was used as a control. Mixtures were loaded into NT.Label Free standard capillaries and measure with 40% MST power. Raw data was integrated and fitted to a binding model using MST analysis software (Version 1.5.41) [58].

### Statistical analysis

Each experiment was performed with at least 3 replicate measurements and presented as mean ± standard deviation. One-way analysis of variance (ANOVA) with Tukey’s HSD was used to determine significant differences for multi-sample experiments with one variable. Two-way ANOVA with Tukey’s HSD was used to determine significant differences for multi-variable analyses.

## Supporting information

S1 Fig SDS treatment-induced autophagy is dependent on CWI kinases. (A) Guy11, Δ*Momck1*, Δ*Momkk1*, and Δ*Momps1* were treated without (CM) or with 0.008% SDS for 5 h, vacuoles with Abs were then quantified. (B) Hyphae of Guy11, Δ*Momck1*, Δ*Momkk1*, and Δ*Momps1*, which were transformed with the *RFP-MoATG8* vector, were treated without or with SDS, and RFP-MoAtg8 localization was observed after staining by CMAC to assess autophagic levels. (C) Cells with vacuole-localized RFP-MoAtg8 were quantified as shown in (B). (D) Degradation of MoSec63-GFP was analyzed using Anti-GFP and Anti-Actin antibodies after 5 h treatment of 1 mg/ml CFW or starvation (MM-N). SDS treatment: treated with 0.008% SDS for 2 h. Data from three independent experiments were used for statistical analysis by two-way ANOVA with Tukey’s HSD.
(TIF)

S2 Fig MoMkk1 phosphorylates MoAtg4. (A and B) Localization of MoMkk1 (A) and MoAtg4 (B) under CM or CFW treatment conditions. MoApe1-RFP was used as a PAS marker. (C) Quantification of cells with PAS-localized MoAtg4 under CM or CFW treatment. Data from three independent experiments were used for statistical analysis by one-way ANOVA with Tukey’s HSD. (D) BiFC observation of MoMkk1 and MoAtg4 after MDC staining. Arrows indicate MDC-stained dot in the cytoplasm. (E) MoAtg4-GFP proteins from Δ*Moatg4*/*MoATG4-GFP*, Δ*Momkk1*/*MoATG4-GFP*, Δ*Moatg1*/*MoATG4-GFP*, and Δ*Moatg1*Δ*Momkk1*/*MoATG4-GFP* strains were purified with anti-GFP beads, and treated with a phosphatase or a phosphatase inhibitor, then normal and Mn^2+^-Phos-tag SDS-PAGE were performed. Phosphorylation was analyzed by Western blot using the anti-GFP antibody. (F) Analysis of MoAtg4 phosphorylation in Δ*Moatg4*/*MoATG4-GFP*, Δ*Momkk1*/*MoATG4-GFP*, Δ*Moatg1*/*MoATG4-GFP* treated with 1 mg/ml CFW, 10 mM DTT, or MM-N for 5 h with no treatment as a control. (G) Phosphorylated residues of MoAtg4 identified by LC-MS/MS analysis or FDIT method. Scale bar: 5 μm.
(TIF)

S3 Fig MoAtg4 phosphorylation is important for conidium germination and appressorium formation. (A-D) Inoculated conidial suspensions to hydrophobic slides, then conidial germination and appressorium formation were observed at 2, 6, 10, and 24 hpi in the dark using a microscope. Different letters indicate statistically significant differences (Duncan’s new multiple range test, *p* < 0.01).
(TIF)

S4 Fig MoMkk1-dependent MoAtg4 phosphorylation is not involved in CWI signaling. (A-C) Growth, conidiation, and pathogenicity analysis of Δ*Moatg4*/3A and Δ*Moatg4*/3D under 400 μg/ml CFW treatment. Conidiation on SDC medium as control. N/A indicates not available. (D) Hyphae of Guy11, Δ*Moatg4*, Δ*Moatg4*/3A, and Δ*Moatg4*/3D strains were stained with 10 μg/ml CFW for 5 min in darkness, and cell wall chitin distribution was observed by confocal microscopy. Scale bar: 5 μm. (E) Quantification of apices with even chitin distribution as shown in (D). (F) Autolysis observation of Guy11, Δ*Moatg4*, Δ*Moatg4*/3A, and Δ*Moatg4*/3D strains on CM plates at 15 dpi. (G) MoMps1 phosphorylation in Δ*Moatg4*/3A and Δ*Moatg4*/3D under 1 mg/ml CFW treatment for 5 h was analyzed by P-P44/42 and P44/42 antibodies. The Δ*Momkk1* strain was used as a positive control for CWI defect and hyphal autolysis. Different letters indicate statistically significant differences (Duncan’s new multiple range test, *p* < 0.01). Data from three independent experiments were used for statistical analysis by one-way ANOVA with Tukey’s HSD.
(TIF)

S5 Fig MoMkk1-dependent MoAtg4 phosphorylation negatively regulates autophagy under cell wall stress. (A) AB observation after MDC staining under CFW treatment. C: cytoplasm. V: vacuole. (B) Quantification of vacuoles with AB as shown in (A). Different letters indicate statistically significant differences (Duncan’s new multiple range test, *p* < 0.01). (C) An amino acid sequence alignment of *M*. *oryzae* MoAtg8 and *S*. *cerevisiae* Atg8. (D) Cleavage assay of MoAtg8-RFP in mycelia following treatment with CFW. (E) MoAtg8-RFP localization was observed at the appressorium stage. (F) Cleavage assay of MoAtg8-RFP at the conidia stage. (G) Co-IP assay to analyze the interaction between MoAtg4^3A^ or MoAtg4^3D^, and MoAtg8 under CFW treatment. (H) Interaction analysis of MoAtg4^3A^ and MoAtg4^3D^ with MoMkk1 by Y2H. CFW treatment: treated with 1 mg/ml CFW for 5 h. Scale bar: 5 μm.
(TIF)

S6 Fig MoAtg1-mediated MoAtg4 phosphorylation is involved in the development and pathogenicity of *M*. *oryzae*. (A) Y2H analysis of MoAtg1 and MoAtg4 interaction. (B) Strain co-expressing MoAtg1-nYFP and MoAtg4-cYFP was treated with MM-N for 3 h, and BiFC signals were observed using a confocal microscope. MoApe1-RFP was used as the PAS marker. Scale bar: 5 μm. (C) *In vitro* phosphorylation analysis of His-MoAtg1 with His-MoAtg4, or His-MoAtg4^2A^, His-MoAtg4^S67A^, His-MoAtg4^S364A^. (D) Identified peptide of MoAtg1-mediated MoAtg4 phosphorylation by LC-MS/MS analysis under MM-N treatment. (E) S364 peptide alignment of *M*. *oryzae* MoAtg4 and yeast Atg4. (F) Conidiation analysis on SDC medium. (G) Virulence analysis of Δ*Moatg4*/2A and Δ*Moatg4*/2D strains. (H) Lesion quantification as shown in (G) by Image J. 2A/2D: replacement of S67 and S364 with A/D to mimic nonphosphorylated/phosphorylated form of MoAtg4 mediated by MoAtg1. Different letters indicate statistically significant differences (Duncan’s new multiple range test, *p* < 0.01).
(TIF)

S7 Fig MoAtg1-mediated MoAtg4 phosphorylation negatively regulates autophagy by inhibiting MoAtg8-PE deconjugation. (A) AB observation after MDC staining. C: cytoplasm. V: vacuole. (B) Quantification of vacuoles with AB as shown in (A). (C) AB observation using TEM. The arrow points to AB. Scale bar: 0.5 μm. (D) Quantification of AB as shown in (C). (E) Guy11, Δ*Moatg4*, Δ*Moatg4*/2A, and Δ*Moatg4*/2D strains, which were transformed with the *RFP*-*MoATG8*^G116^ vector, were treated with MM-N for 3 h. The subcellular localization was observed after staining by CMAC that analyzes MoAtg8-PE deconjugation. Linescan graph (indicated by yellow arrows) and statistical analysis of RFP-MoAtg8^G116^ localization were displayed below. (F) Conidia were inoculated to hydrophobic slides for 8 h, RFP-MoAtg8^G116^ localization was then observed and linescan graph (indicated by yellow arrows) of RFP-MoAtg8^G116^ localization were displayed. Protein levels, estimated using 6 M urea SDS-PAGE gel electrophoresis, were used to analyze MoAtg8-PE deconjugation at the appressorium development stage. (G) Y2H analysis for binding abilities of MoAtg4, MoAtg4^2A^, and MoAtg4^2D^ to MoAtg8. (H) Δ*Moatg4*/*MoATG4*, Δ*Moatg4*/2A, and Δ*Moatg4*/2D strains expressing RFP-MoAtg8^G116^ were treated with MM-N for 3 h; proteins then were extracted for co-IP assay and Western blot analysis using anti-RFP and anti-GFP antibodies. Proteins from Δ*Moatg4* strain co-expressing GFP and RFP-MoAtg8^G116^ were used as a control. Scale bar: 5 μm. Different letters indicate statistically significant differences (Duncan’s new multiple range test, *p* < 0.01).
(TIF)

S8 Fig MoMkk1-dependent MoAtg4 phosphorylation is not involved in MoAtg8-PE deconjugation. (A and B) MoAtg8-PE deconjugation analysis in Δ*Moatg4*/3A, Δ*Moatg4*/3D (A), Δ*Momck1*, Δ*Momkk1*, and Δ*Momps1* (B) strains expressing RFP-MoAtg8^G116^. Scale bar: 5 μm.
(TIF)

S9 Fig MoMkk1- and MoAtg1-dependent MoAtg4 phosphorylation events cooperate to regulate development and pathogenicity. (A) Conidiation analysis of Δ*Moatg4*/5A and Δ*Moatg4*/5D strains. (B) Pathogenicity analysis on 2-week-old rice seedlings. (C) Quantification of diseased leaf areas as shown in (B) by Image J. (D) The severity of rice blasts was evaluated by quantitative PCR as shown in (B). (E) Close observations and statistical analysis of IH growth in rice leaf sheaths. (F and G) *In vitro* phosphorylation analysis of GST-MoMkk1 with His-MoAtg4 or His-MoAtg4^2D^, His-MoAtg1 with His-MoAtg4 or His-MoAtg4^3D^, and His-MoAtg4 with GST-MoMkk1 or GST-MoMkk1^S115D^ (GST-S115D). (H) MoMkk1 phosphorylation in Δ*Moatg4*/3A and Δ*Moatg4*/3D under 10 mM DTT treatment for 5 h was analyzed. (I) Cleavage assay of MoAtg8 under 1 mg/ml CFW treatment for 5 h. Different letters indicate statistically significant differences (Duncan’s new multiple range test, *p* < 0.01). Data from three independent experiments were used for statistical analysis by one-way ANOVA with Tukey’s HSD.
(TIF)

## References

[1] Sánchez-Wandelmer J, Kriegenburg F, Rohringer S, Schuschnig M, Gómez-Sánchez R. Atg4 proteolytic activity can be inhibited by Atg1 phosphorylation. Nat Commun. 2017;8(1):295. doi: 10.1038/s41467-017-00302-3 . 28821724 PMC5562703

[2] Yin Z, Chen C, Yang J, Feng W, Liu X, Zuo R, et al. Histone acetyltransferase MoHat1 acetylates autophagy-related proteins MoAtg3 and MoAtg9 to orchestrate functional appressorium formation and pathogenicity in *Magnaporthe oryzae*. Autophagy. 2019;15(7):1234–1257. doi: 10.1080/15548627.2019.1580104 . 30776962 PMC6613890

[3] Zaffagnini G, Martens S. Mechanisms of selective autophagy. J Mol Biol. 2016;428(9 Pt A):1714–1724. doi: 10.1016/j.jmb.2016.02.004 . 26876603 PMC4871809

[4] Liu XH, Lu JP, Zhang L, Dong B, Min H, Lin FC. Involvement of a *Magnaporthe grisea* serine/threonine kinase gene, *MgATG1*, in appressorium turgor and pathogenesis. Eukaryot Cell. 2007;6(6):997–1005. doi: 10.1128/ec.00011-07 . 17416896 PMC1951528

[5] Liu TB, Liu XH, Lu JP, Zhang L, Min H, Lin FC. The cysteine protease MoAtg4 interacts with MoAtg8 and is required for differentiation and pathogenesis in *Magnaporthe oryzae*. Autophagy. 2010;6(1):74–85. doi: 10.4161/auto.6.1.10438 . 19923912

[6] Kershaw MJ, Talbot NJ. Genome-wide functional analysis reveals that infection-associated fungal autophagy is necessary for rice blast disease. Proc Natl Acad Sci U S A. 2009;106(37):15967–15972. doi: 10.1073/pnas.0901477106 . 19717456 PMC2747227

[7] Deng YZ, Ramos-Pamplona M, Naqvi NI. Autophagy-assisted glycogen catabolism regulates asexual differentiation in *Magnaporthe oryzae*. Autophagy. 2009;5(1):33–43. doi: 10.4161/auto.5.1.7175 . 19115483

[8] Ichimura Y, Kirisako T, Takao T, Satomi Y, Shimonishi Y, Ishihara N, et al. A ubiquitin-like system mediates protein lipidation. Nature. 2000;408(6811):488–492. doi: 10.1038/35044114 . 11100732

[9] Yoshimoto K, Hanaoka H, Sato S, Kato T, Tabata S, Noda T, et al. Processing of ATG8s, ubiquitin-like proteins, and their deconjugation by ATG4s are essential for plant autophagy. Plant Cell. 2004;16(11):2967–2983. doi: 10.1105/tpc.104.025395 . 15494556 PMC527192

[10] Hanada T, Noda NN, Satomi Y, Ichimura Y, Fujioka Y, Takao T, et al. The Atg12-Atg5 conjugate has a novel E3-like activity for protein lipidation in autophagy. J Biol Chem. 2007;282(52):37298–38302. doi: 10.1074/jbc.C700195200 . 17986448

[11] Jotwani A, Richerson DN, Motta I, Julca-Zevallos O, Melia TJ. Approaches to the study of Atg8-mediated membrane dynamics *in vitro*. Methods Cell Biol. 2012;108:93–116. doi: 10.1016/b978-0-12-386487-1.00005-5 . 22325599

[12] Kaufmann A, Beier V, Franquelim HG, Wollert T. Molecular mechanism of autophagic membrane-scaffold assembly and disassembly. Cell. 2014;156(3):469–481. doi: 10.1016/j.cell.2013.12.022 . 24485455

[13] Sakoh-Nakatogawa M, Matoba K, Asai E, Kirisako H, Ishii J, Noda NN, et al. Atg12-Atg5 conjugate enhances E2 activity of Atg3 by rearranging its catalytic site. Nat Struct Mol Biol. 2013;20(4):433–439. doi: 10.1038/nsmb.2527 . 23503366

[14] Feng W, Yin Z, Wu H, Liu P, Liu X, Liu M, et al. Balancing of the mitotic exit network and cell wall integrity signaling governs the development and pathogenicity in *Magnaporthe oryzae*. PLoS Pathog. 2021;17(1):e1009080. doi: 10.1371/journal.ppat.1009080 . 33411855 PMC7817018

[15] Bacete L, Hamann T. The role of mechanoperception in plant cell wall integrity maintenance. Plants (Basel). 2020;9(5):574. doi: 10.3390/plants9050574 . 32369932 PMC7285163

[16] Yin Z, Feng W, Chen C, Xu J, Li Y, Yang L, et al. Shedding light on autophagy coordinating with cell wall integrity signaling to govern pathogenicity of *Magnaporthe oryzae*. Autophagy. 2020;16(5):900–916. doi: 10.1080/15548627.2019.1644075 . 31313634 PMC7144863

[17] Levin DE. Regulation of cell wall biogenesis in *Saccharomyces cerevisiae*: the cell wall integrity signaling pathway. Genetics. 2011;189(4):1145–1175. doi: 10.1534/genetics.111.128264 . 22174182 PMC3241422

[18] Kock C, Dufrêne YF, Heinisch JJ. Up against the wall: is yeast cell wall integrity ensured by mechanosensing in plasma membrane microdomains? Appl Environ Microbiol. 2015;81(3):806–811. doi: 10.1128/AEM.03273-14 . 25398859 PMC4292499

[19] Levin DE. Cell wall integrity signaling in *Saccharomyces cerevisiae*. Microbiol Mol Biol Rev. 2005;69(2):262–291. doi: 10.1128/mmbr.69.2.262-291.2005 . 15944456 PMC1197416

[20] Mey G, Held K, Scheffer J, Tenberge KB, Tudzynski P. CPMK2, an SLT2-homologous mitogen-activated protein (MAP) kinase, is essential for pathogenesis of *Claviceps purpurea* on rye: evidence for a second conserved pathogenesis-related MAP kinase cascade in phytopathogenic fungi. Mol Microbiol. 2002;46(2):305–318. doi: 10.1046/j.1365-2958.2002.03133.x . 12406210

[21] Yun Y, Liu Z, Zhang J, Shim WB, Chen Y, Ma Z. The MAPKK FgMkk1 of *Fusarium graminearum* regulates vegetative differentiation, multiple stress response, and virulence via the cell wall integrity and high-osmolarity glycerol signaling pathways. Environ Microbiol. 2014;16(7):2023–2037. doi: 10.1111/1462-2920.12334 . 24237706

[22] Yin Z, Tang W, Wang J, Liu X, Yang L, Gao C, et al. Phosphodiesterase MoPdeH targets MoMck1 of the conserved mitogen-activated protein (MAP) kinase signalling pathway to regulate cell wall integrity in rice blast fungus *Magnaporthe oryzae*. Mol Plant Pathol. 2016;17(5):654–668. doi: 10.1111/mpp.12317 . 27193947 PMC6638318

[23] Penn TJ, Wood ME, Soanes DM, Csukai M, Corran AJ, Talbot NJ. Protein kinase C is essential for viability of the rice blast fungus *Magnaporthe oryzae*. Mol Microbiol. 2015;98(3):403–419. doi: 10.1111/mmi.13132 . 26192090 PMC4791171

[24] Jeon J, Goh J, Yoo S, Chi MH, Choi J, Rho HS, et al. A putative MAP kinase kinase kinase, *MCK1*, is required for cell wall integrity and pathogenicity of the rice blast fungus, *Magnaporthe oryzae*. Mol Plant Microbe Interact. 2008;21(5):525–534. doi: 10.1094/mpmi-21-5-0525 . 18393612

[25] Xu JR, Staiger CJ, Hamer JE. Inactivation of the mitogen-activated protein kinase Mps1 from the rice blast fungus prevents penetration of host cells but allows activation of plant defense responses. Proc Natl Acad Sci U S A. 1998;95(21):12713–12718. doi: 10.1073/pnas.95.21.12713 . 9770551 PMC22896

[26] Mao K, Wang K, Zhao M, Xu T, Klionsky DJ. Two MAPK-signaling pathways are required for mitophagy in *Saccharomyces cerevisiae*. J Cell Biol. 2011;193(4):755–767. doi: 10.1083/jcb.201102092 . 21576396 PMC3166859

[27] Mao K, Klionsky DJ. MAPKs regulate mitophagy in *Saccharomyces cerevisiae*. Autophagy. 2011;7(12):1564–1565. doi: 10.4161/auto.7.12.17971 . 22024747 PMC3327622

[28] Manjithaya R, Jain S, Farré JC, Subramani S. A yeast MAPK cascade regulates pexophagy but not other autophagy pathways. J Cell Biol. 2010;189(2):303–310. doi: 10.1083/jcb.200909154 . 20385774 PMC2856896

[29] Corral-Ramos C, Roca MG, Di Pietro A, Roncero MI, Ruiz-Roldán C. Autophagy contributes to regulation of nuclear dynamics during vegetative growth and hyphal fusion in *Fusarium oxysporum*. Autophagy. 2015;11(1):131–144. doi: 10.4161/15548627.2014.994413 . 25560310 PMC4507430

[30] Josefsen L, Droce A, Sondergaard TE, Sørensen JL, Bormann J, Schäfer W, et al. Autophagy provides nutrients for nonassimilating fungal structures and is necessary for plant colonization but not for infection in the necrotrophic plant pathogen *Fusarium graminearum*. Autophagy. 2012;8(3):326–337. doi: 10.4161/auto.18705 . 22240663

[31] Veneault-Fourrey C, Barooah M, Egan M, Wakley G, Talbot NJ. Autophagic fungal cell death is necessary for infection by the rice blast fungus. Science. 2006;312(5773):580–583. doi: 10.1126/science.1124550 . 16645096

[32] Wei YY, Liang S, Zhang YR, Lu JP, Lin FC, Liu XH. MoSec61β, the beta subunit of Sec61, is involved in fungal development and pathogenicity, plant immunity, and ER-phagy in *Magnaporthe oryzae*. Virulence. 2020;11(1):1685–1700. doi: 10.1080/21505594.2020.1848983 . 33200669 PMC7714445

[33] Wang S, Li QP, Wang J, Yan Y, Zhang GL, Yan Y, et al. YR36/WKS1-mediated phosphorylation of PsbO, an extrinsic member of photosystem II, inhibits photosynthesis and confers stripe rust resistance in wheat. Mol Plant. 2019;12(12):1639–1650. doi: 10.1016/j.molp.2019.10.005 . 31622682

[34] Yu R, Shen X, Liu M, Liu X, Yin Z, Li X, et al. The rice blast fungus MoRgs1 functioning in cAMP signaling and pathogenicity is regulated by casein kinase MoCk2 phosphorylation and modulated by membrane protein MoEmc2. PLoS Pathog. 2021;17(6):e1009657. doi: 10.1371/journal.ppat.1009657 . 34133468 PMC8208561

[35] Liu X, Gao Y, Guo Z, Wang N, Wegner A, Wang J, et al. MoIug4 is a novel secreted effector promoting rice blast by counteracting host OsAHL1-regulated ethylene gene transcription. New Phytol. 2022;235(3):1163–1178. doi: 10.1111/nph.18169 . 35451078 PMC11164540

[36] Guo Z, Liu X, Wang N, Mo P, Shen J, Liu M, et al. Membrane component ergosterol builds a platform for promoting effector secretion and virulence in *Magnaporthe oryzae*. New Phytol. 2023;237(3):930–943. doi: 10.1111/nph.18575 . 36300785

[37] Pengo N, Agrotis A, Prak K, Jones J. A reversible phospho-switch mediated by ULK1 regulates the activity of autophagy protease ATG4B. Nat Commun. 2017;8(1):294. doi: 10.1038/s41467-017-00303-2 . 28821708 PMC5562857

[38] Nair U, Yen WL, Mari M, Cao Y, Xie Z, Baba M, et al. A role for Atg8-PE deconjugation in autophagosome biogenesis. Autophagy. 2012;8(5):780–793. doi: 10.4161/auto.19385 . 22622160 PMC3378420

[39] Li B, Song S, Wei X, Tang G, Wang C. Activation of microlipophagy during early infection of insect hosts by *Metarhizium robertsii*. Autophagy. 2022;18(3):608–623. doi: 10.1080/15548627.2021.1943179 . 34130590 PMC9037503

[40] Klionsky DJ, Abdel-Aziz AK, Abdelfatah S, Abdellatif M, Abdoli A, Abel S, et al. Guidelines for the use and interpretation of assays for monitoring autophagy (4th edition)(1). Autophagy. 2021;17(1):1–382. doi: 10.1080/15548627.2020.1797280 . 33634751 PMC7996087

[41] Rajendran P, Alzahrani AM, Hanieh HN, Kumar SA, Ben Ammar R, Rengarajan T, et al. Autophagy and senescence: a new insight in selected human diseases. J Cell Physiol. 2019;234(12):21485–21492. doi: 10.1002/jcp.28895 . 31144309

[42] Kang C, Avery L. To be or not to be, the level of autophagy is the question: dual roles of autophagy in the survival response to starvation. Autophagy. 2008;4(1):82–84. doi: 10.4161/auto.5154 . 17952023 PMC4440895

[43] Feng X, Zhang H, Meng L, Song H, Zhou Q, Qu C, et al. Hypoxia-induced acetylation of PAK1 enhances autophagy and promotes brain tumorigenesis via phosphorylating ATG5. Autophagy. 2021;17(3):723–742. doi: 10.1080/15548627.2020.1731266 . 32186433 PMC8032228

[44] Yu ZQ, Ni T, Hong B, Wang HY, Jiang FJ, Zou S, et al. Dual roles of Atg8-PE deconjugation by Atg4 in autophagy. Autophagy. 2012;8(6):883–892. doi: 10.4161/auto.19652 . 22652539 PMC3427254

[45] Huang T, Kim CK, Alvarez AA, Pangeni RP, Wan X, Song X, et al. MST4 phosphorylation of ATG4B regulates autophagic activity, tumorigenicity, and radioresistance in Glioblastoma. Cancer Cell. 2017;32(6):840–855.e8. doi: 10.1016/j.ccell.2017.11.005 . 29232556 PMC5734934

[46] Kirisako T, Ichimura Y, Okada H, Kabeya Y, Mizushima N, Yoshimori T, et al. The reversible modification regulates the membrane-binding state of Apg8/Aut7 essential for autophagy and the cytoplasm to vacuole targeting pathway. J Cell Biol. 2000;151(2):263–276. doi: 10.1083/jcb.151.2.263 . 11038174 PMC2192639

[47] Liu X, Zhou Q, Guo Z, Liu P, Shen L, Chai N, et al. A self-balancing circuit centered on MoOsm1 kinase governs adaptive responses to host-derived ROS in *Magnaporthe oryzae*. eLife. 2020;9:e61605. doi: 10.7554/eLife.61605 . 33275098 PMC7717906

[48] Li X, Zhong K, Yin Z, Hu J, Wang W, Li L, et al. The seven transmembrane domain protein MoRgs7 functions in surface perception and undergoes coronin MoCrn1-dependent endocytosis in complex with Galpha subunit MoMagA to promote cAMP signaling and appressorium formation in *Magnaporthe oryzae*. PLoS Pathog. 2019;15(2):e1007382. doi: 10.1371/journal.ppat.1007382 . 30802274 PMC6405168

[49] Kim J, Kundu M, Viollet B, Guan KL. AMPK and mTOR regulate autophagy through direct phosphorylation of Ulk1. Nat Cell Biol. 2011;13(2):132–141. doi: 10.1038/ncb2152 . 21258367 PMC3987946

[50] Suzuki K, Kubota Y, Sekito T, Ohsumi Y. Hierarchy of Atg proteins in pre-autophagosomal structure organization. Genes Cells. 2007;12(2):209–218. doi: 10.1111/j.1365-2443.2007.01050.x . 17295840

[51] Kamada Y, Yoshino K, Kondo C, Kawamata T, Oshiro N, Yonezawa K, et al. Tor directly controls the Atg1 kinase complex to regulate autophagy. Mol Cell Biol. 2010;30(4):1049–1058. doi: 10.1128/MCB.01344-09 . 19995911 PMC2815578

[52] Liu M, Hu J, Zhang A, Dai Y, Chen W, He Y, et al. Auxilin-like protein MoSwa2 promotes effector secretion and virulence as a clathrin uncoating factor in the rice blast fungus *Magnaporthe oryzae*. New Phytol. 2021;230(2):720–736. doi: 10.1111/nph.17181 . 33423301 PMC8048681

[53] Qian B, Su X, Ye Z, Liu X, Liu M, Shen D, et al. MoErv29 promotes apoplastic effector secretion contributing to virulence of the rice blast fungus *Magnaporthe oryzae*. New Phytol. 2022;233(3):1289–1302. doi: 10.1111/nph.17851 . 34761375 PMC8738142

[54] Zhong K, Li X, Le X, Kong X, Zhang H, Zheng X, et al. MoDnm1 dynamin mediating peroxisomal and mitochondrial fission in complex with MoFis1 and MoMdv1 is important for development of functional appressorium in *Magnaporthe oryzae*. PLoS Pathog. 2016;12(8):e1005823. doi: 10.1371/journal.ppat.1005823 . 27556292 PMC4996533

[55] Liu M, Zhang S, Hu J, Sun W, Padilla J, He Y, et al. Phosphorylation-guarded light-harvesting complex II contributes to broad-spectrum blast resistance in rice. Proc Natl Acad Sci U S A. 2019;116(35):17572–17577. doi: 10.1073/pnas.1905123116 . 31405986 PMC6717248

[56] Qian B, Su X, Ye Z, Liu X, Liu M, Zhang H, et al. MoErv14 mediates the intracellular transport of cell membrane receptors to govern the appressorial formation and pathogenicity of *Magnaporthe oryzae*. PLoS Pathog. 2023;19(4):e1011251. doi: 10.1371/journal.ppat.1011251 . 37011084 PMC10101639

[57] Jin X, Gou JY. A rapid and cost-effective fluorescence detection in tube (FDIT) method to analyze protein phosphorylation. Plant Methods. 2016;12:43. doi: 10.1186/s13007-016-0143-5 . 27822293 PMC5094037

[58] Hu J, Liu M, Zhang A, Dai Y, Chen W, Chen F, et al. Co-evolved plant and blast fungus ascorbate oxidases orchestrate the redox state of host apoplast to modulate rice immunity. Mol Plant. 2022;15(8):1347–1366. doi: 10.1016/j.molp.2022.07.001 . 35799449 PMC11163382

