# Supplementary figures and images for: Autophagy and cell wall integrity pathways coordinately regulate the development and pathogenicity through MoAtg4 phosphorylation in Magnaporthe oryzae
Source: PLoS Pathog. 2024 Jan 30;20(1):e1011988. doi: 10.1371/journal.ppat.1011988 (PMC10857709; doi:10.1371/journal.ppat.1011988)

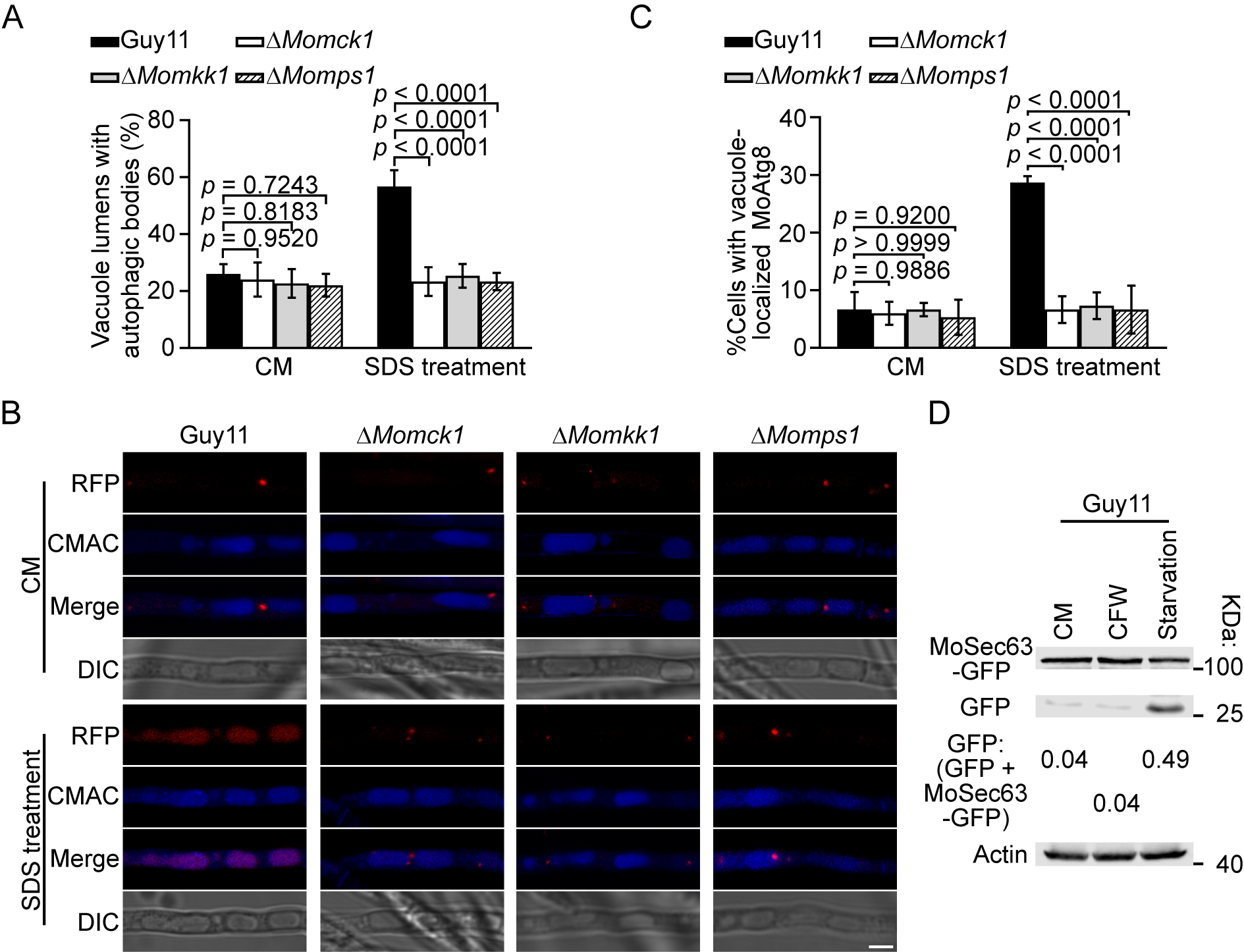

Supplement: S1 Fig — (A) Guy11, ΔMomck1, ΔMomkk1, and ΔMomps1 were treated without (CM) or with 0.008% SDS for 5 h, vacuoles with Abs were then quantified. (B) Hyphae of Guy11, ΔMomck1, ΔMomkk1, and ΔMomps1, which were transformed with the RFP-MoATG8 vector, were treated without or with SDS, and RFP-MoAtg8 localization was observed after staining by CMAC to assess autophagic levels. (C) Cells with vacuole-localized RFP-MoAtg8 were quantified as shown in (B). (D) Degradation of MoSec63-GFP was analyzed using Anti-GFP and Anti-Actin antibodies after 5 h treatment of 1 mg/ml CFW or starvation (MM-N). SDS treatment: treated with 0.008% SDS for 2 h. Data from three independent experiments were used for statistical analysis by two-way ANOVA with Tukey’s HSD. (TIF) [file ppat.1011988.s001.tif]

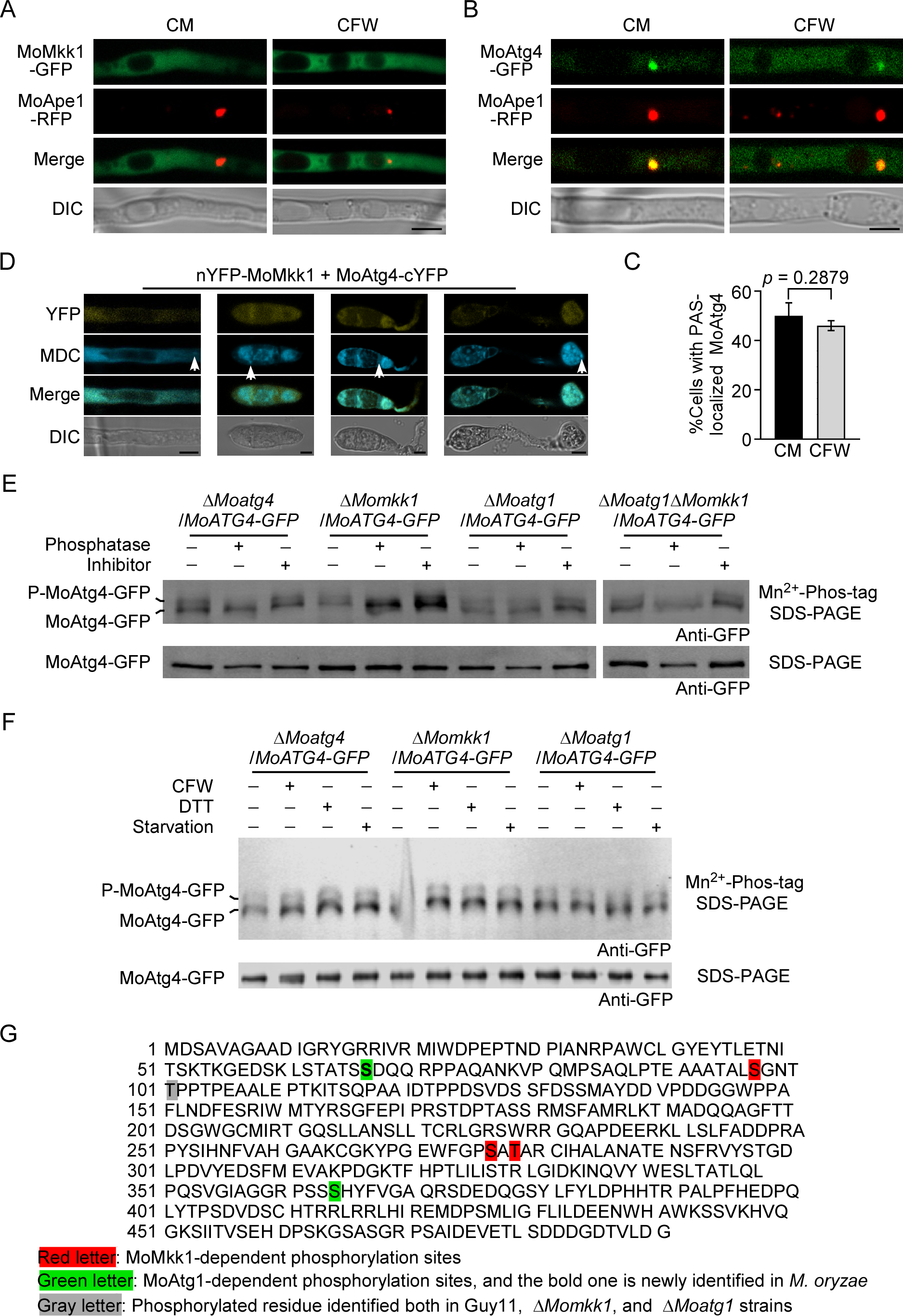

Supplement: S2 Fig — (A and B) Localization of MoMkk1 (A) and MoAtg4 (B) under CM or CFW treatment conditions. MoApe1-RFP was used as a PAS marker. (C) Quantification of cells with PAS-localized MoAtg4 under CM or CFW treatment. Data from three independent experiments were used for statistical analysis by one-way ANOVA with Tukey’s HSD. (D) BiFC observation of MoMkk1 and MoAtg4 after MDC staining. Arrows indicate MDC-stained dot in the cytoplasm. (E) MoAtg4-GFP proteins from ΔMoatg4/MoATG4-GFP, ΔMomkk1/MoATG4-GFP, ΔMoatg1/MoATG4-GFP, and ΔMoatg1ΔMomkk1/MoATG4-GFP strains were purified with anti-GFP beads, and treated with a phosphatase or a phosphatase inhibitor, then normal and Mn2+-Phos-tag SDS-PAGE were performed. Phosphorylation was analyzed by Western blot using the anti-GFP antibody. (F) Analysis of MoAtg4 phosphorylation in ΔMoatg4/MoATG4-GFP, ΔMomkk1/MoATG4-GFP, ΔMoatg1/MoATG4-GFP treated with 1 mg/ml CFW, 10 mM DTT, or MM-N for 5 h with no treatment as a control. (G) Phosphorylated residues of MoAtg4 identified by LC-MS/MS analysis or FDIT method. Scale bar: 5 μm. (TIF) [file ppat.1011988.s002.tif]

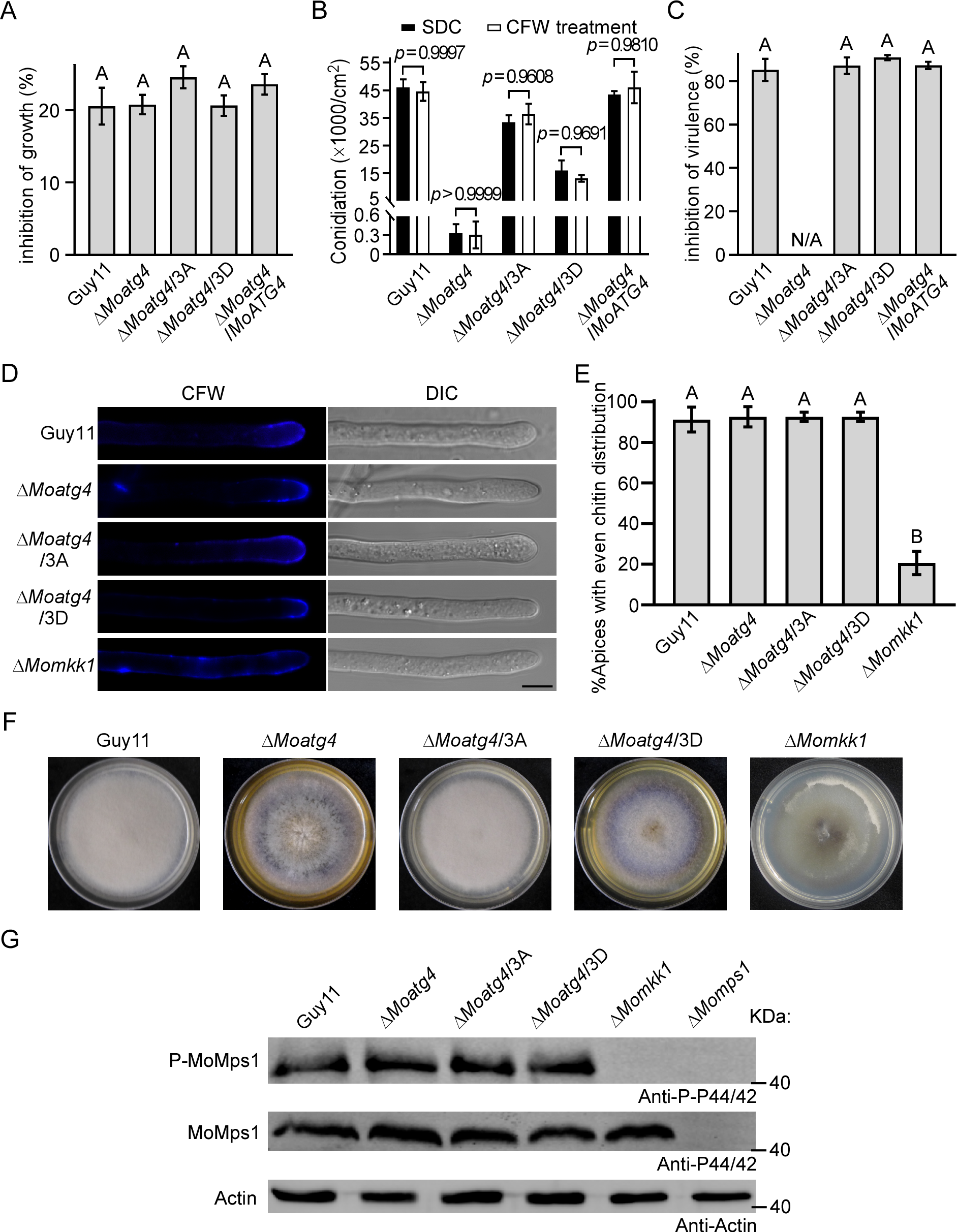

Supplement: S4 Fig — (A-C) Growth, conidiation, and pathogenicity analysis of ΔMoatg4/3A and ΔMoatg4/3D under 400 μg/ml CFW treatment. Conidiation on SDC medium as control. N/A indicates not available. (D) Hyphae of Guy11, ΔMoatg4, ΔMoatg4/3A, and ΔMoatg4/3D strains were stained with 10 μg/ml CFW for 5 min in darkness, and cell wall chitin distribution was observed by confocal microscopy. Scale bar: 5 μm. (E) Quantification of apices with even chitin distribution as shown in (D). (F) Autolysis observation of Guy11, ΔMoatg4, ΔMoatg4/3A, and ΔMoatg4/3D strains on CM plates at 15 dpi. (G) MoMps1 phosphorylation in ΔMoatg4/3A and ΔMoatg4/3D under 1 mg/ml CFW treatment for 5 h was analyzed by P-P44/42 and P44/42 antibodies. The ΔMomkk1 strain was used as a positive control for CWI defect and hyphal autolysis. Different letters indicate statistically significant differences (Duncan’s new multiple range test, p < 0.01). Data from three independent experiments were used for statistical analysis by one-way ANOVA with Tukey’s HSD. (TIF) [file ppat.1011988.s004.tif]

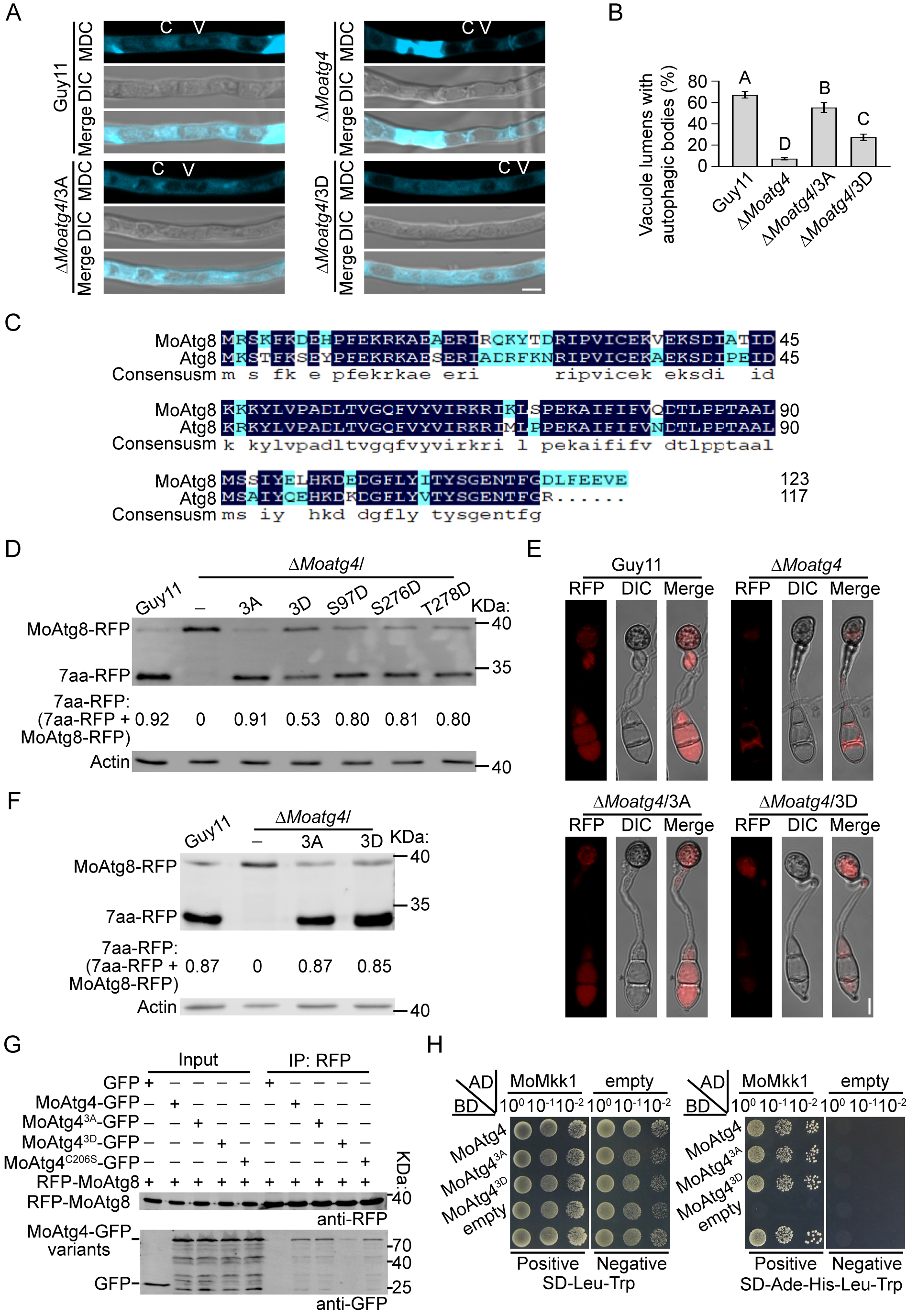

Supplement: S5 Fig — (A) AB observation after MDC staining under CFW treatment. C: cytoplasm. V: vacuole. (B) Quantification of vacuoles with AB as shown in (A). Different letters indicate statistically significant differences (Duncan’s new multiple range test, p < 0.01). (C) An amino acid sequence alignment of M. oryzae MoAtg8 and S. cerevisiae Atg8. (D) Cleavage assay of MoAtg8-RFP in mycelia following treatment with CFW. (E) MoAtg8-RFP localization was observed at the appressorium stage. (F) Cleavage assay of MoAtg8-RFP at the conidia stage. (G) Co-IP assay to analyze the interaction between MoAtg43A or MoAtg43D, and MoAtg8 under CFW treatment. (H) Interaction analysis of MoAtg43A and MoAtg43D with MoMkk1 by Y2H. CFW treatment: treated with 1 mg/ml CFW for 5 h. Scale bar: 5 μm. (TIF) [file ppat.1011988.s005.tif]

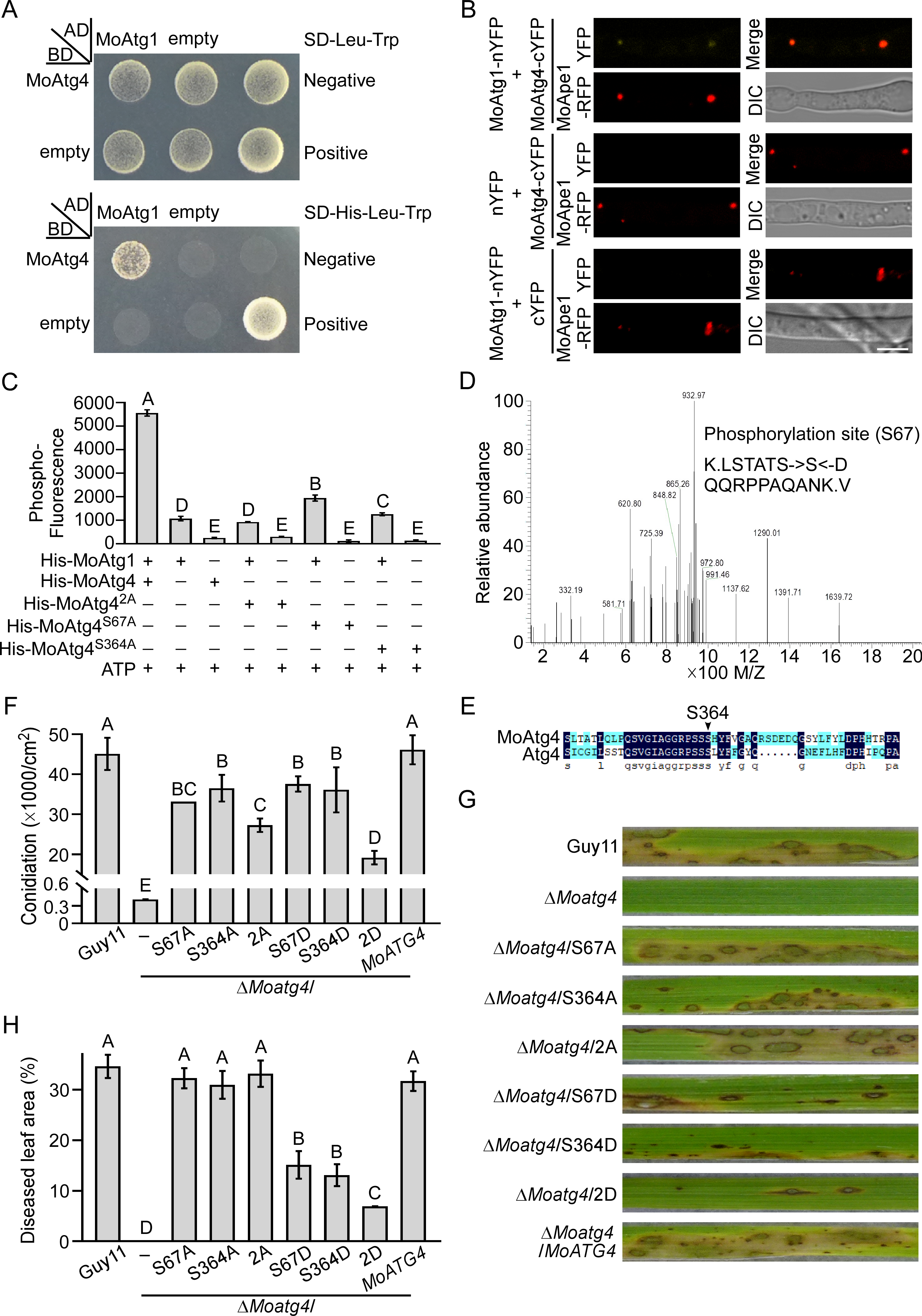

Supplement: S6 Fig — (A) Y2H analysis of MoAtg1 and MoAtg4 interaction. (B) Strain co-expressing MoAtg1-nYFP and MoAtg4-cYFP was treated with MM-N for 3 h, and BiFC signals were observed using a confocal microscope. MoApe1-RFP was used as the PAS marker. Scale bar: 5 μm. (C) In vitro phosphorylation analysis of His-MoAtg1 with His-MoAtg4, or His-MoAtg42A, His-MoAtg4S67A, His-MoAtg4S364A. (D) Identified peptide of MoAtg1-mediated MoAtg4 phosphorylation by LC-MS/MS analysis under MM-N treatment. (E) S364 peptide alignment of M. oryzae MoAtg4 and yeast Atg4. (F) Conidiation analysis on SDC medium. (G) Virulence analysis of ΔMoatg4/2A and ΔMoatg4/2D strains. (H) Lesion quantification as shown in (G) by Image J. 2A/2D: replacement of S67 and S364 with A/D to mimic nonphosphorylated/phosphorylated form of MoAtg4 mediated by MoAtg1. Different letters indicate statistically significant differences (Duncan’s new multiple range test, p < 0.01). (TIF) [file ppat.1011988.s006.tif]

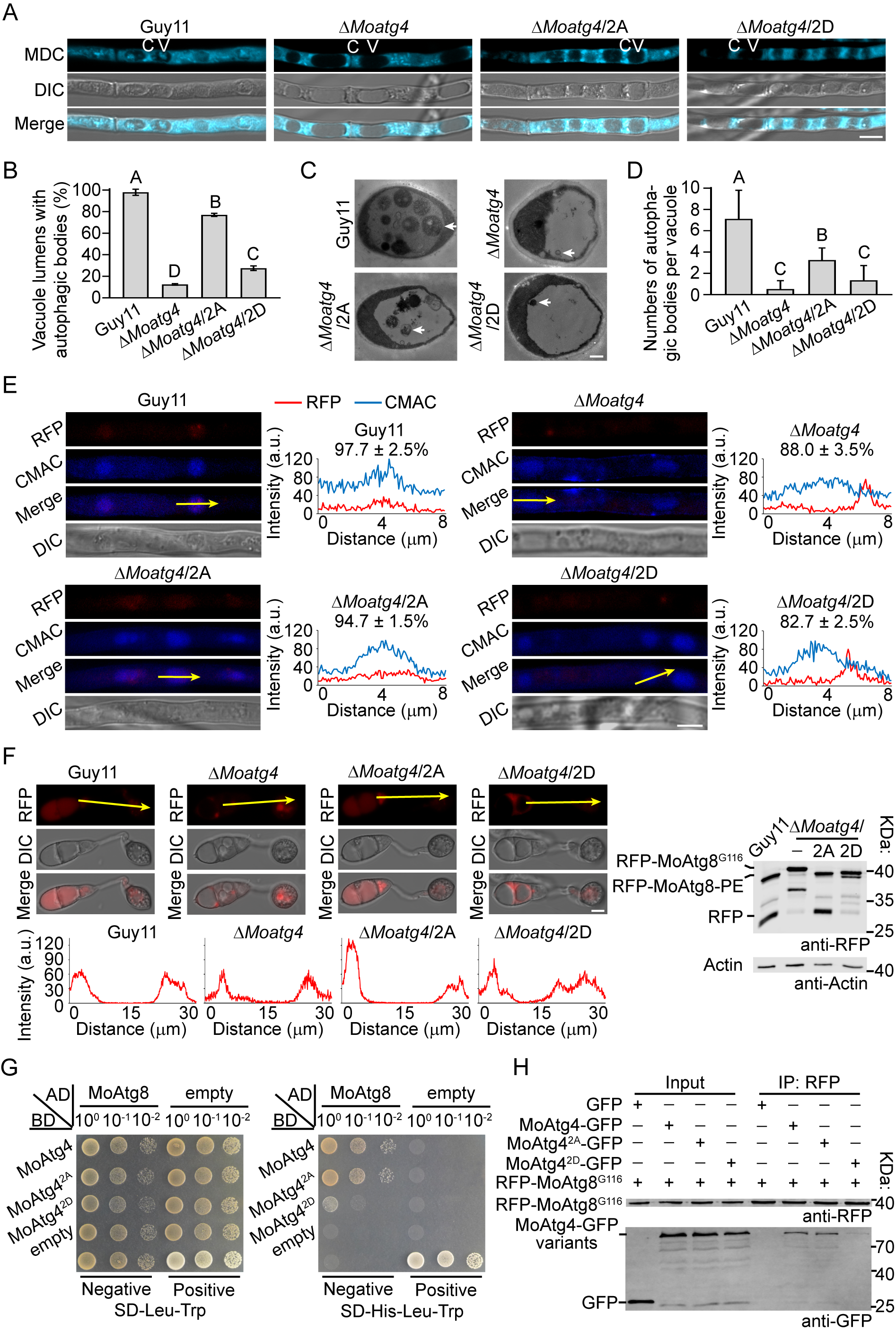

Supplement: S7 Fig — (A) AB observation after MDC staining. C: cytoplasm. V: vacuole. (B) Quantification of vacuoles with AB as shown in (A). (C) AB observation using TEM. The arrow points to AB. Scale bar: 0.5 μm. (D) Quantification of AB as shown in (C). (E) Guy11, ΔMoatg4, ΔMoatg4/2A, and ΔMoatg4/2D strains, which were transformed with the RFP-MoATG8G116 vector, were treated with MM-N for 3 h. The subcellular localization was observed after staining by CMAC that analyzes MoAtg8-PE deconjugation. Linescan graph (indicated by yellow arrows) and statistical analysis of RFP-MoAtg8G116 localization were displayed below. (F) Conidia were inoculated to hydrophobic slides for 8 h, RFP-MoAtg8G116 localization was then observed and linescan graph (indicated by yellow arrows) of RFP-MoAtg8G116 localization were displayed. Protein levels, estimated using 6 M urea SDS-PAGE gel electrophoresis, were used to analyze MoAtg8-PE deconjugation at the appressorium development stage. (G) Y2H analysis for binding abilities of MoAtg4, MoAtg42A, and MoAtg42D to MoAtg8. (H) ΔMoatg4/MoATG4, ΔMoatg4/2A, and ΔMoatg4/2D strains expressing RFP-MoAtg8G116 were treated with MM-N for 3 h; proteins then were extracted for co-IP assay and Western blot analysis using anti-RFP and anti-GFP antibodies. Proteins from ΔMoatg4 strain co-expressing GFP and RFP-MoAtg8G116 were used as a control. Scale bar: 5 μm. Different letters indicate statistically significant differences (Duncan’s new multiple range test, p < 0.01). (TIF) [file ppat.1011988.s007.tif]

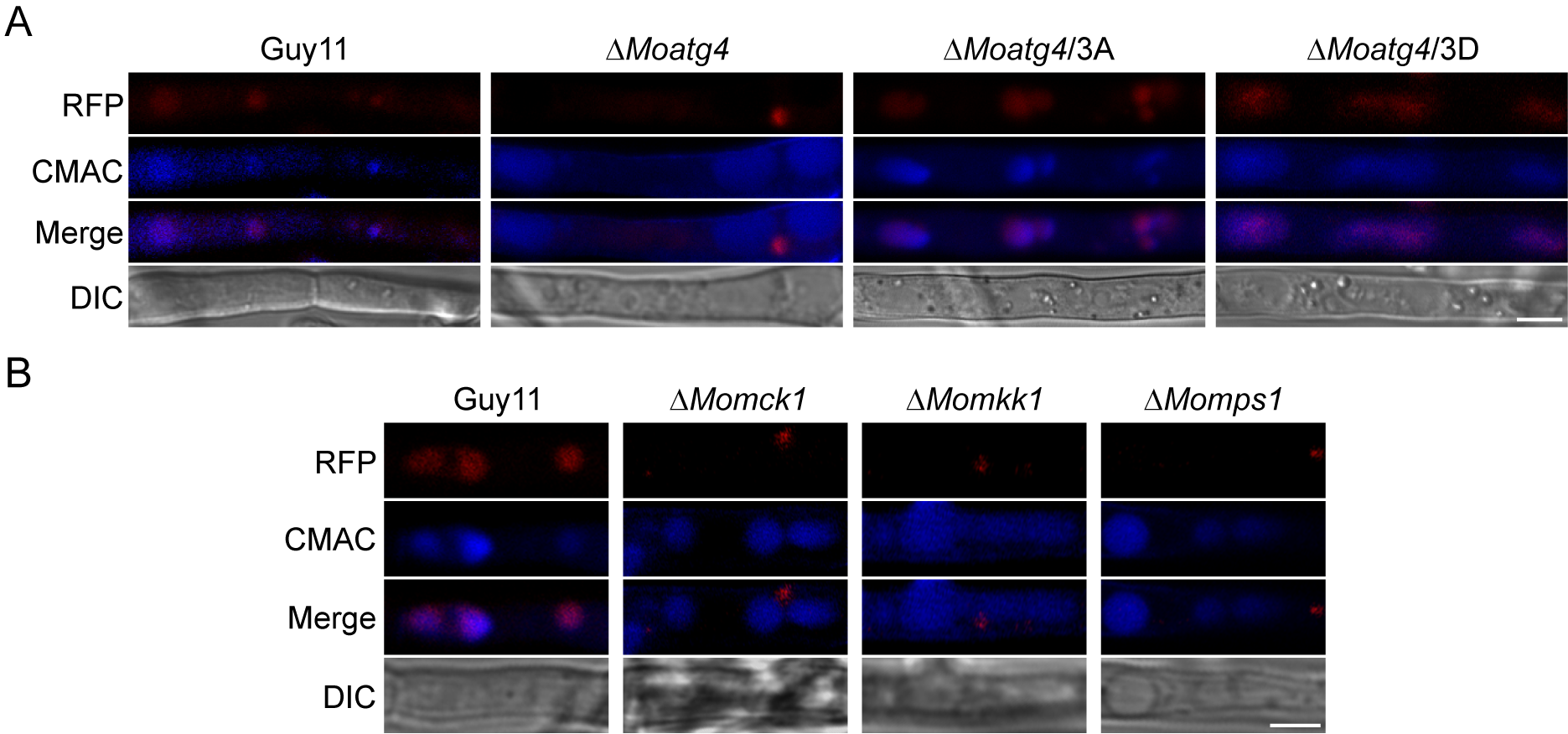

Supplement: S8 Fig — (A and B) MoAtg8-PE deconjugation analysis in ΔMoatg4/3A, ΔMoatg4/3D (A), ΔMomck1, ΔMomkk1, and ΔMomps1 (B) strains expressing RFP-MoAtg8G116. Scale bar: 5 μm. (TIF) [file ppat.1011988.s008.tif]

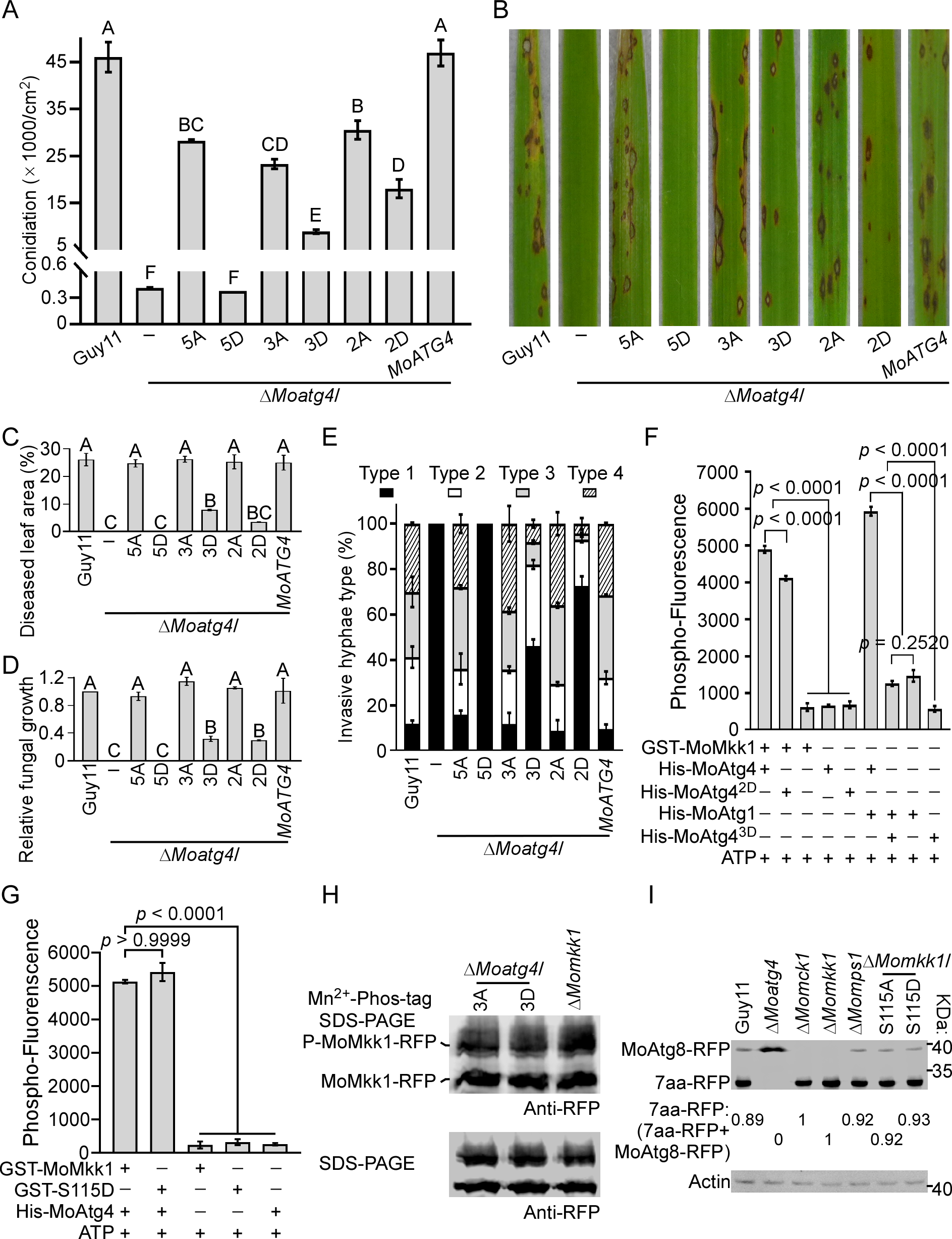

Supplement: S9 Fig — (A) Conidiation analysis of ΔMoatg4/5A and ΔMoatg4/5D strains. (B) Pathogenicity analysis on 2-week-old rice seedlings. (C) Quantification of diseased leaf areas as shown in (B) by Image J. (D) The severity of rice blasts was evaluated by quantitative PCR as shown in (B). (E) Close observations and statistical analysis of IH growth in rice leaf sheaths. (F and G) In vitro phosphorylation analysis of GST-MoMkk1 with His-MoAtg4 or His-MoAtg42D, His-MoAtg1 with His-MoAtg4 or His-MoAtg43D, and His-MoAtg4 with GST-MoMkk1 or GST-MoMkk1S115D (GST-S115D). (H) MoMkk1 phosphorylation in ΔMoatg4/3A and ΔMoatg4/3D under 10 mM DTT treatment for 5 h was analyzed. (I) Cleavage assay of MoAtg8 under 1 mg/ml CFW treatment for 5 h. Different letters indicate statistically significant differences (Duncan’s new multiple range test, p < 0.01). Data from three independent experiments were used for statistical analysis by one-way ANOVA with Tukey’s HSD. (TIF) [file ppat.1011988.s009.tif]
